# Dose-Response Transcranial Electrical Stimulation Study Design: A Well-Controlled Adaptive Seamless Bayesian Method to Illuminate Negative Valence Role in Tinnitus Perception

**DOI:** 10.3389/fnhum.2022.811550

**Published:** 2022-05-12

**Authors:** Iman Ghodratitoostani, Oilson A. Gonzatto, Zahra Vaziri, Alexandre C. B. Delbem, Bahador Makkiabadi, Abhishek Datta, Chris Thomas, Miguel A. Hyppolito, Antonio C. D. Santos, Francisco Louzada, João Pereira Leite

**Affiliations:** ^1^Neurocognitive Engineering Laboratory, Center for Engineering Applied to Health, Institute of Mathematics and Computer Science, University of São Paulo, São Carlos, Brazil; ^2^Institute of Mathematics and Computer Science, University of São Paulo, São Carlos, Brazil; ^3^Department of Neuroscience and Behavior, Faculty of Medicine of Ribeirão Preto, University of São Paulo, Ribeirao Preto, Brazil; ^4^Research Center for Biomedical Technologies and Robotics, Institute for Advanced Medical Technologies, Tehran, Iran; ^5^Department of Medical Physics and Biomedical Engineering, School of Medicine, Tehran University of Medical Sciences, Tehran, Iran; ^6^Soterix Medical, New York, NY, United States; ^7^Department of Ophthalmology, Otorhinolaryngology, Head and Neck Surgery, Ribeirão Preto Medical School, University of São Paulo, Ribeirao Preto, Brazil

**Keywords:** evaluative conditional learning, neurofunctional tinnitus model, positive emotion induction, high definition-transcranial direct current stimulation, loudness misperception correction, adaptive seamless study design, bayesian method, dose-response relationship

## Abstract

The use of transcranial Electrical Stimulation (tES) in the modulation of cognitive brain functions to improve neuropsychiatric conditions has extensively increased over the decades. tES techniques have also raised new challenges associated with study design, stimulation protocol, functional specificity, and dose-response relationship. In this paper, we addressed challenges through the emerging methodology to investigate the dose-response relationship of High Definition-transcranial Direct Current Stimulation (HD tDCS), identifying the role of negative valence in tinnitus perception. In light of the neurofunctional testable framework and tES application, hypotheses were formulated to measure clinical and surrogate endpoints. We posited that conscious pairing adequately pleasant stimuli with tinnitus perception results in correction of the loudness misperception and would be reinforced by concurrent active HD-tDCS on the left Dorsolateral Prefrontal Cortex (dlPFC). The dose-response relationship between HD-tDCS specificity and the loudness perception is also modeled. We conducted a double-blind, randomized crossover pilot study with six recruited tinnitus patients. Accrued data was utilized to design a well-controlled adaptive seamless Bayesian dose-response study. The sample size (*n* = 47, for 90% power and 95% confidence) and optimum interims were anticipated for adaptive decision-making about efficacy, safety, and single session dose parameters. Furthermore, preliminary pilot study results were sufficient to show a significant difference (90% power, 99% confidence) within the longitudinally detected self-report tinnitus loudness between before and under positive emotion induction. This study demonstrated a research methodology used to improve emotion regulation in tinnitus patients. In the projected method, positive emotion induction is essential for promoting functional targeting under HD-tDCS anatomical specificity to indicate the efficacy and facilitate the dose-finding process. The continuous updating of prior knowledge about efficacy and dose during the exploratory stage adapts the anticipated dose-response model. Consequently, the effective dose range to make superiority neuromodulation in correcting loudness misperception of tinnitus will be redefined. Highly effective dose adapts the study to a standard randomized trial and transforms it into the confirmatory stage in which active HD-tDCS protocol is compared with a sham trial (placebo-like). Establishing the HD-tDCS intervention protocols relying on this novel method provides reliable evidence for regulatory agencies to approve or reject the efficacy and safety. Furthermore, this paper supports a technical report for designing multimodality data-driven complementary investigations in emotion regulation, including EEG-driven neuro markers, Stroop-driven attention biases, and neuroimaging-driven brain network dynamics.

## 1. Introduction

Tinnitus is a Conscious Attended-Awareness Perception (CAAP) of sourceless sound. Several studies have reported that auditory phantom perception affects 30% of the general population worldwide (Mills et al., 1986; Heller, 2003; Coelho et al., 2007; Savastano, 2007). It remains unclear why only 17% of the affected subjects experience bothersome when perceiving tinnitus (Axelsson and Ringdahl, 1989). Several cognitive and behavioral theoretical models have attempted to unravel the impact of psychological factors and associated mechanisms in triggering or mitigating tinnitus distress (Jastreboff, 1990; Hallam et al., 2004; Zenner and Zalaman, 2004; Andersson and McKenna, 2006; Zenner et al., 2006; McKenna et al., 2014; Ghodratitoostani et al., 2016a,b). Hallam et al. (2004) proposed that failure in habituation to tinnitus causes increased awareness because of negative appraisal and emotional significance. Subsequently, classical conditioning was proposed as the principal mechanism behind the aversive emotional states of tinnitus (Jastreboff, 1990). Later, Zenner et al. (2006) postulated that tinnitus sensitization develops when perceiving sound is classified as noxious, fear-inducing, unpredictable, and might cause a sense of deficiency in coping, and helplessness (Zenner and Zalaman, 2004; Zenner et al., 2006). McKenna et al. (2014), in their study, documented that cognitive misinterpretation of the tinnitus results in distress and physiological arousal, leading to distorted perception from sensory input.

Different attentional paradigms have revealed significant impairments in selective attention among tinnitus patients (Jastreboff, 1990; Baguley et al., 2013; Roberts et al., 2013; McKenna et al., 2014; Li et al., 2015). Trevis et al. (2016) reported that patients with chronic tinnitus had weaker performance in cognitive tasks in a silent room with repetitive background noise than the healthy controls. Emotional Stroop Task (EST) is one of the earliest methods developed for assessing attentional bias to emotional or concern-relevant information Gross (2013) and Davidson et al. (2000). Recently, Ghodratitoostani et al. (2016b) proposed the Neurofunctional Tinnitus Model (NfTM) and highlighted that the CAAP of tinnitus is essential for causing bothersome. NfTM classifies tinnitus patients into two stages: A) “Neutral stage”: perceiving tinnitus without distress reaction and B) “Clinical distress stage”: experiencing distress reaction because of the corresponding negative valence when the tinnitus is perceived (Ghodratitoostani et al., 2016a,b). Valence represents emotional states varying along a continuum from positive to negative feelings with a neutral midpoint (Bradley and Lang, 1994). Tinnitus-related valence progressively becomes negative through the Evaluative Conditional Learning (ECL) mechanism wherein repeated pairing of neutral tinnitus conditioned with similar or different negative stimuli unconditioned develops negative valence (De Houwer et al., 2001; Ghodratitoostani et al., 2016b). On the other hand, negative appraisals such as “*The noise makes my life unbearable,” “it will drive me crazy,” or “it will overwhelm me”* (Handscomb et al., 2017) intermittently reinforce the cognitive value of tinnitus. Appraisal and ECL mechanisms drive tinnitus-related cognitive-emotional value and lead to preferential attention allocation to the sound and prolonged tinnitus perception [13]. Contrarily, NfTM has postulated that the CAAP of tinnitus concurrently presenting positively-valenced stimuli might improve negative valence and might lead to perceiving tinnitus less frequently and with a lower level of distress (Ghodratitoostani et al., 2016b). Cognitive functions suggested in NfTM can also be embodied in the emotion regulation process model (Gross, 1998, 2013) of tinnitus, which predicts that the tinnitus loudness misperception may be associated with the negative valence and selective attention. NfTM also postulates that continuous evaluation of tinnitus valence, comparing this valence with those of other sensory and auditory inputs, and monitoring persistent perception occurs in the prefrontal cortex (Ghodratitoostani et al., 2016b). More specifically, the dlPFC revealed associations between auditory attention (Breit et al., 2004) and the processing of emotional information (Steele and Lawrie, 2004; Jacob et al., 2014). Neuroimaging studies on emotion have shown enhanced activity in dlPFC, especially on the left hemisphere. An association between positive mood orientation and positive-stimuli processing was also noticed (Davidson et al., 2000). NfTM proposed that anodal tDCS modulatory effect at the left dlPFC reinforces the induced positive emotional stimulation and reduces tinnitus-related negative valence. Using tDCS for relieving tinnitus-related negative valence (Ghodratitoostani et al., 2016b) depends on the effect of electrical stimulation on the active brain networks, that reinforce or decline the excitability underneath the anode or cathode, respectively (Rahman et al., 2013). The impact of down-regulating negative emotional processing was documented with the application of anodal tDCS on dlPFC, but not with the cathodal stimulation in some studies (Nitsche and Paulus, 2000; Fregni et al., 2005; Lang et al., 2005). tDCS specificity generally depends on functional and anatomical targeting mechanisms. Targeting refers to subthreshold modulatory-effects on particular functionally-active brain regions regarding stimulation (anodal or cathodal) montages. Functional targeting in tDCS applications may occur through preferentially modulating persistent function on an active brain network (Jackson et al., 2016). Functional targeting may also occur due to applied bias to different synaptic inputs (Bikson and Rahman, 2013). Anatomical targeting is the focal neuromodulation on specific brain regions by delivering the desired electrical dose achieved only by regulating the tDCS parameters (Peterchev et al., 2012). The dose-response relationship can be measured after assessing the response of the associated functionally-active networks affected via induced current circuitry, even though the brain targeted region is involved in multiple tasks during tDCS stimulation (Bikson and Rahman, 2013). HD-tDCS employs multielectrode montages to improve the anatomical targeting by enhancing the focality of current flow (Dmochowski et al., 2011; Bikson and Rahman, 2013). The electrical-dose of tDCS emerges from stimulation device settings that affect the electrical field generated in the targeted brain areas. Dose parameters include stimulation waveform (direct current-DC), intensity, duration, polarity, montage, number of sessions; number, type, and shape of electrodes (Peterchev et al., 2012). The dose can be calculated by multiplying current-intensity by time (duration of stimulation) formulated in Equation (1) below


(1) $$\begin{array}{l} \text{Dose}=\text{Intensity}\times \text{Time}, \end{array}$$

while electrode type, waveform, polarity, applied intensity, and montage remain constant (Jamil et al., 2017). Individual anatomical variations in head size, skull, skin thickness, and color have been shown to affect Dose to the targeted area (Thomas et al., 2019). The mechanism of current-intensity, duration, electrode size, and montage affecting tDCS-related responses remains inconsistent (Monte-Silva et al., 2013; Esmaeilpour et al., 2018; Agboada et al., 2019; Jamil et al., 2020). Moreover, determining sufficient stimulating sessions and intervals pose potential challenges associated with clinical treatment planning while considering the individual diversity in brain anatomy, connectivity, and emerging functions (Goldsworthy and Hordacre, 2017; Jamil et al., 2017; Thomas et al., 2019). In this study, we employed the self-assessment Tinnitus Loudness Questionnaire (TLQ) scale as a surrogate endpoint to verify the correction of loudness misperception in correlation with ongoing modulations of tinnitus negative-valence. TLQ was regularly collected under applying HD-tDCS concurrent with positive emotion induction (through presenting positively-valenced pictures) against only positive emotion induction. Moreover, ongoing recording variations of TLQ rating concurrent with HD-tDCS helped investigate the dose-response relationship. We primarily hypothesized that the conscious pairing of adequate pleasant visual stimuli concurrent with tinnitus perception results in correction of loudness misperception. Second, Active HD-tDCS on the left dlPFC facilitates the correction of loudness misperception. So, the dose-response relationship between HD-tDCS specificity and the correction of loudness misperception is proposed. We designed a well-controlled, two-stage, seamless, adaptive double-blind, and randomized crossover trial, and conducted a pilot study on six tinnitus patients for sample size calculation, clinical endpoint (minimally clinical efficacy) optimization, and dose selection in a single-session stimulation. We applied conventional and adaptive approaches for seamless statistical Bayesian design for analytical comparison. Meanwhile, the designed protocol promotes data-driven investigation on EEG-driven neuro markers, Stroop-driven attentional bias, and neuroimaging-driven brain network connectivity-dynamics. These are explained in the latter part of this paper.

## 2. Materials and Methods

### 2.1. Inclusion/Exclusion Criteria

A total of six patients, referred to the Specialized Center of Otorhinolaryngology and Speech Therapy, Medical Complex Hospital of Ribeirão Preto, Medical School-University of São Paulo, (HCRP- FMRP-USP), Brazil (HCRP No. 55716616.1.1001.5440), were recruited for the pilot study. Literate patients with constant bilateral subjective tinnitus, normal hearing, or utmost moderate sensorineural hearing loss, normal color vision, and no history of psychoactive medication were included. Patients with pulsatile tinnitus, Meniere's disease, otosclerosis, chronic headache, and other neurological disorders such as brain tumors, and those treated for mental or central nervous system disorders were excluded. All recruited patients signed written informed consent.

### 2.2. Audiological and Tinnitus Psychoacoustic Evaluation

All the recruited participants underwent Pure Tone Audiometry (125–16,000 Hz) for hearing assessment at recruiting time. Psychoacoustic evaluation of tinnitus was performed at recruiting time and before and after each session, including, Laterality, Similarity, Pitch Matching test (PMT), Hearing Threshold Level (HTL), Loudness Match Test (LMT), Minimal Masking Level (MML), and loudness discomfort level (LDL). We explored Complete procedures in Supplementary Materials.

### 2.3. Baseline Assessment and Instruction

In the screening session, patients filled in Tinnitus Sample Case Tinnitus Sample Case History Questionnaire (TSCHQ), Tinnitus Handicap Inventory (THI), Tinnitus Impairment Questionnaire (TBF-12), Tinnitus Severity (TS), Major Depression Inventory (MDI), the Portuguese Short version of the State Trait Anxiety Inventroy Small Questions (STAI-S6), and the WHO-Quality of Life instrument (WHO-QoL). All the patients were adequately trained for adhering to proper instructions about the given tasks of the experiment. The tasks were as under.

1. Not moving their head and body,
2. Focusing on their tinnitus sound while enjoying the pictures,
3. Answering TLQ, which was frequently presented on LCD as “*scale your tinnitus loudness”*, by pressing a key from ***F1*** to ***F10*** on the modified keyboard, and
4. Accurately adjusting fingers on the corresponding colored keys during the EST, ignoring the meaning of the words and responding to their color as fast as possible. Additionally, they were asked to avoid drinking coffee, alcoholic beverages, and cigarette smoking at least 24 h before the experimental sessions.

## 3. Study Design

An adaptive seamless observational crossover, randomized, double-blind study was designed in the following three sessions:

1. Active-Control or positive emotion induction (PEI) via the presentation of a set of validated positively-valenced pictures,
2. Anodal HD-tDCS_4×1_ (20 min, 2 mA with 30 s Ramp-up/ ramp-down) concurrent with PEI, and
3. placebo-like effect i.e., sham stimulation concurrent with PEI (**Figure 5D**).

An audiologist assisted and accompanied the patients through the experiment. The audiologist instantly before and after each session was responsible for the evaluation of the clinical tinnitus to measure tinnitus type, side, and pitch to specify its psychoacoustic parameters. The patients were tested to identify the best match to the perceived frequency of their tinnitus, followed by assessments of tinnitus-related parameters. Such parameters included the hearing HTL, LMT, MML, and LDL, which took 5–15 min only. The patients sat on a fixed pneumatic-armchair in a dark and quiet experiment room in front of a 40-inch LCD at 185 cm distance. Chair height was calibrated individually to ensure that the patients' eyes were at the same altitude from the LCD center. Clinical space was depicted as a 3-Dimensional simulation in **Figures 3A–F**.

### 3.1. Main Experiment Questionnaires Battery

Patients were requested to fill up questionnaires, including THI, TBF-12, MDI, STAI-S6, Clinical Global Impression, Mini Sleep Questionnaire (MSQ), and TS questionnaires. The latter was obtained both before and after each session.

#### 3.1.1. Patient Preparation

Before each session, the EEG Cap-HydroCel EGI-Net was soaked in a solution of 12 mg potassium chloride (KCL) and 10 mL baby shampoo in 1-liter water for 5 min. The maximum circumference was measured for selecting the correct EGI-Net size; the vertex (CZ) was marked on the patient's head. HD-tDCS_4*X*1_ electrode holders were mounted between the EGI-Net elastomers at appropriate places as defined by the head model (**Figure 5A**). The channel gains were measured to confirm whether all impedances of all the sensors were low enough (<50 *KΩ* in EGI-Net machine). After data acquisition, EGI-Net was washed with tap water to clean the conductive solution and gel used for EEG and HD-tDCS_4×1_.

### 3.2. Functional Targeting: The Region of Interest Left-Dorsolateral Prefrontal Cortex

dlPFC is believed to be associated with auditory attention (Breit et al., 2004), auditory processing (Ostrem and Starr, 2008), and emotional processing (Davidson et al., 2000). Neuroimaging investigations revealed the key role of dlPFC in the positive mood (Davidson et al., 2000), emotional processing (Herrington et al., 2005; Rosa et al., 2017), and attentional processing of emotional information (Steele and Lawrie, 2004; Jacob et al., 2014). Furthermore, according to the brain asymmetry model in emotional processing, the left hemisphere prevails over positive emotions; whereas the right hemisphere dominates negative ones (Canli et al., 1998; Alves et al., 2008; Mondino et al., 2015). In line with the Valence Theory within the side-lateralized activity, Vanderhasselt et al. (2013) proposed that the anodal tDCS of left-dlPFC increases neural activity in the left hemisphere and leads to preferential cognitive control for positive information (Vanderhasselt et al., 2013). Non-invasive Brain Stimulation (NIBS) on the left-dlPFC of healthy individuals unveiled modulatory effects on emotional processing. The patients perceived adverse stimuli less negative (Pena-Gomez et al., 2011), enhanced positive stimuli (Nitsche et al., 2012), weakened perception and attention toward negative stimuli (d'Alfonso et al., 2000; De Raedt et al., 2010), and increased positive information retrieval (Mondino et al., 2015) as compared to sham stimulation groups. In contrast, NIBS over right dlPFC showed more identification and attention to negative stimuli and less cognitive control upon negative stimuli (d'Alfonso et al., 2000; De Raedt et al., 2010), though no effect on mood change was expressed (Mondino et al., 2015). Herrington et al. (2005) observed that pleasant words triggered higher activity on the left side dlPFC than on the right one. Furthermore, EEG and functional Magnetic Resonance Imaging (fMRI) studies illustrated that high levels of baseline activity on the left prefrontal cortex had brightened the prospects of suppressing negative emotions (Jackson et al., 2000, 2003; Weissman and Hirsch, 2000; Ochsner et al., 2002).

### 3.3. High Definition- Transcranial Direct Current Stimulation

**Figure 1:**
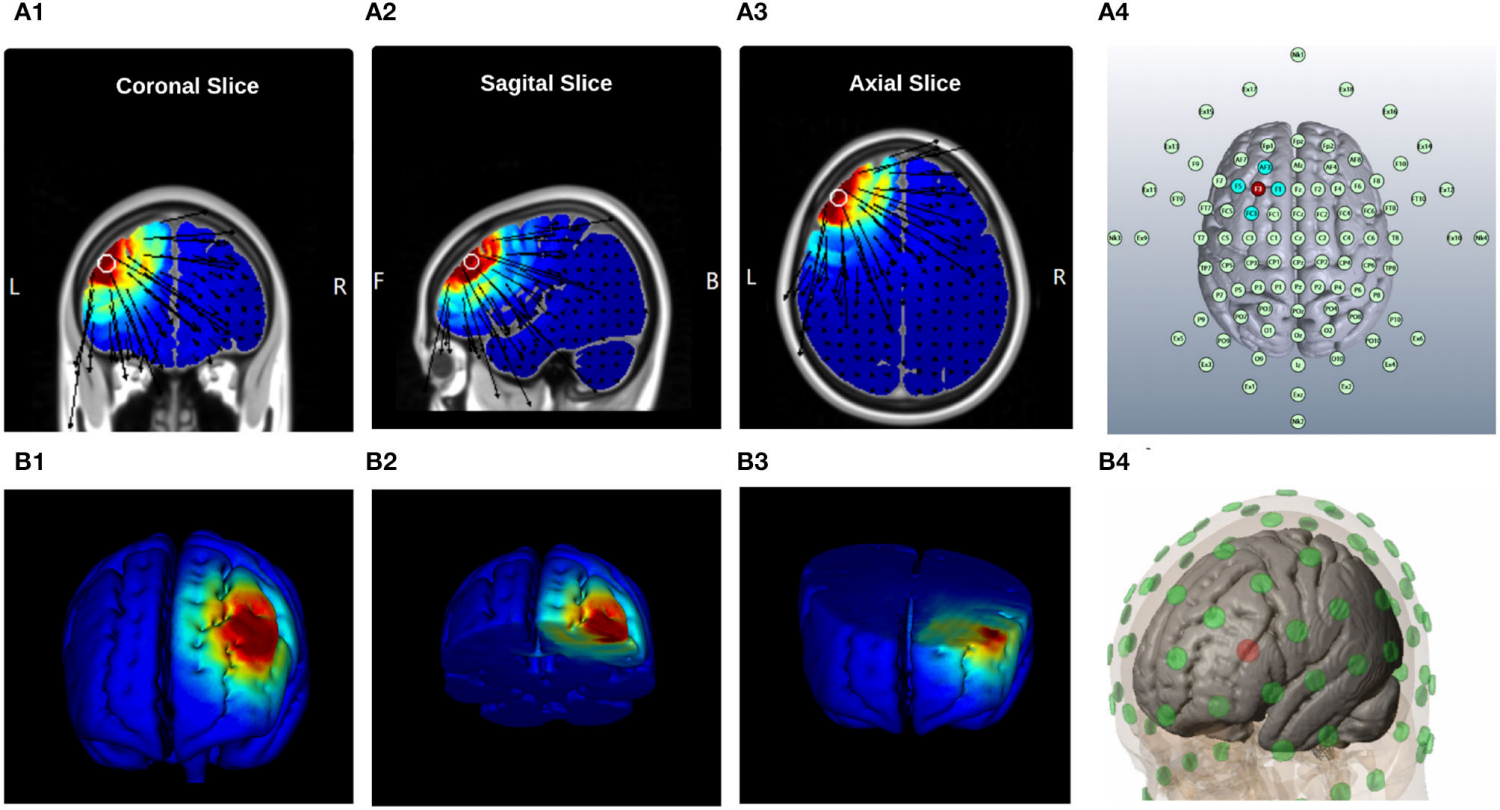
Current Distribution Model, **(A)** 2D Brain Cross-sections, and *MNI position* = [ −42, 36, 35 ] **(A1)** Coronal Slice [56], **(A2)** Sagital Slice [49], **(A3)** Axial Slice [75], and **(A4)** Electrodes layout, “EEG 10-10 system”| Anode: *F*_3_, Cathodes: *F*_1_, *AF*_3_, *AC*_3_, and *F*_5_; **(B)** 3D Brain Cross-sections, **(B1)** Full Brain, **(B2)** Top Brain sections, plane-cut: angle by Y[−34.4], angle by X[3.6], and distance from center[7.7]; **(B3)** Bottom Brain section, plane-cut: angle by Y[97.1], angle by X[5.2], and distance from center[−22.6]; and **(B4)** Electrodes layout, “MNI Atlas 93-electrodes head model,” Anode: *F*_3_, Cathodes: *F*_1_, *AF*_3_, *AC*_3_, and *F*_5_.

A battery-driven current source 1 × 1 DC-Stimulator (Soterix Medical, NY, USA) and a 4 × 1 distributor (Soterix Medical, NY, USA) were administered to deliver 2 mA, HD-tDCS for 20 min with a 30 s ramp up and 30 s ramp down. High-definition gel-based electrodes were used to increase anatomical focality in comparison to conventional electrode pads (Nitsche et al., 2007). According to the head model designed for anodal stimulation of the left-dlPFC (As illustrated in Figure 1) and also the international 10-10 EEG system (Jurcak et al., 2007), the center electrode was placed on F3 with a 2 mA current set. The four cathode-electrodes were placed over F1, F5, AF3, and FC3 and the circuit was closed uniformly dividing total current among the four cathodes placed approximately 3.5 cm away from the anode (Figure 1A4). Such stimulation electrodes were mounted on a 256-channel EEG-Net.

The sham stimulation was performed for 20-min with the same electrodes montage to generate the placebo-like effect. It started and finished with a 30s ramp-up instantly followed by a 30s ramp-down, but insignificant current delivery in between was documented. In this way, the patients experienced the same sensations as that by active HD-tDCS and were kept blind to the intervention (placebo-like effect).

### 3.4. Head Model for HD-tDCS

The brain anatomy (different folding patterns in the cortex, the volume of cerebrospinal fluid, and skull thickness) can considerably influence the current distribution within the head between the electrodes. This variability in current flow among subjects needs personalized head models to ensure anatomical focality in transcranial stimulation (Thomas et al., 2019)s. HD-Targets software (Soterix Medical, New York, USA) was employed to find the optimal electrode placement for the anodal stimulation of the left-dlPFC. We adapted a previously developed finite element (FE) model to further analyze the effect of HD-tDCS_4×1_ electrode montage on the cortical current flow (Datta, 2012). The head model was derived from the classical average brain atlas (ICBM-152; Montreal Neurological Institute, Canada) using a combination of probabilistic segmentation routine, tissue probability map, and a custom segmentation correction script. The template was initially segmented into six tissue categories (scalp, skull, cerebrospinal fluid, gray matter, white matter, and air cavities) (Huang et al., 2013, 2016). Areas representing Brodmann areas (BA) 9 and 46 were manually demarcated in Scan IP (Simpleware Ltd, Exeter, UK) through structures implicated in the tinnitus pathophysiology to analyze current flow patterns. Finally, any outstanding errors in tissue masked continuity were corrected manually. The Gel-based electrodes (12 mm diameter) were imported as computer-aided design (CAD) models and incorporated onto the segmented data to mimic the clinically used montage, anode electrode at F3, and the cathode electrodes at AF3, F1, FC3, and F5. From this segmented dataset, a volumetric mesh was generated and exported to a FE solver (COMSOL Multiphysics 4.3, COMSOL Inc., MA, USA). The following isotropic electrical conductivities (in S/m) were assigned: Scalp − 0.465; skull − 0.01; cerebrospinal fluid − 1.65; gray matter 0 0.276; white matter − 0.126; air 01*e* − 15; gel 0 0.3; electrode 0 5.8*e*7 (Datta, 2012). Since BA 9 and 46 regions are derived from the cerebral cortex, they were assigned the gray matter's tissue conductivity.

### 3.5. Finite Elements Model of the HD-tDCS Montage

**Figure 2:**
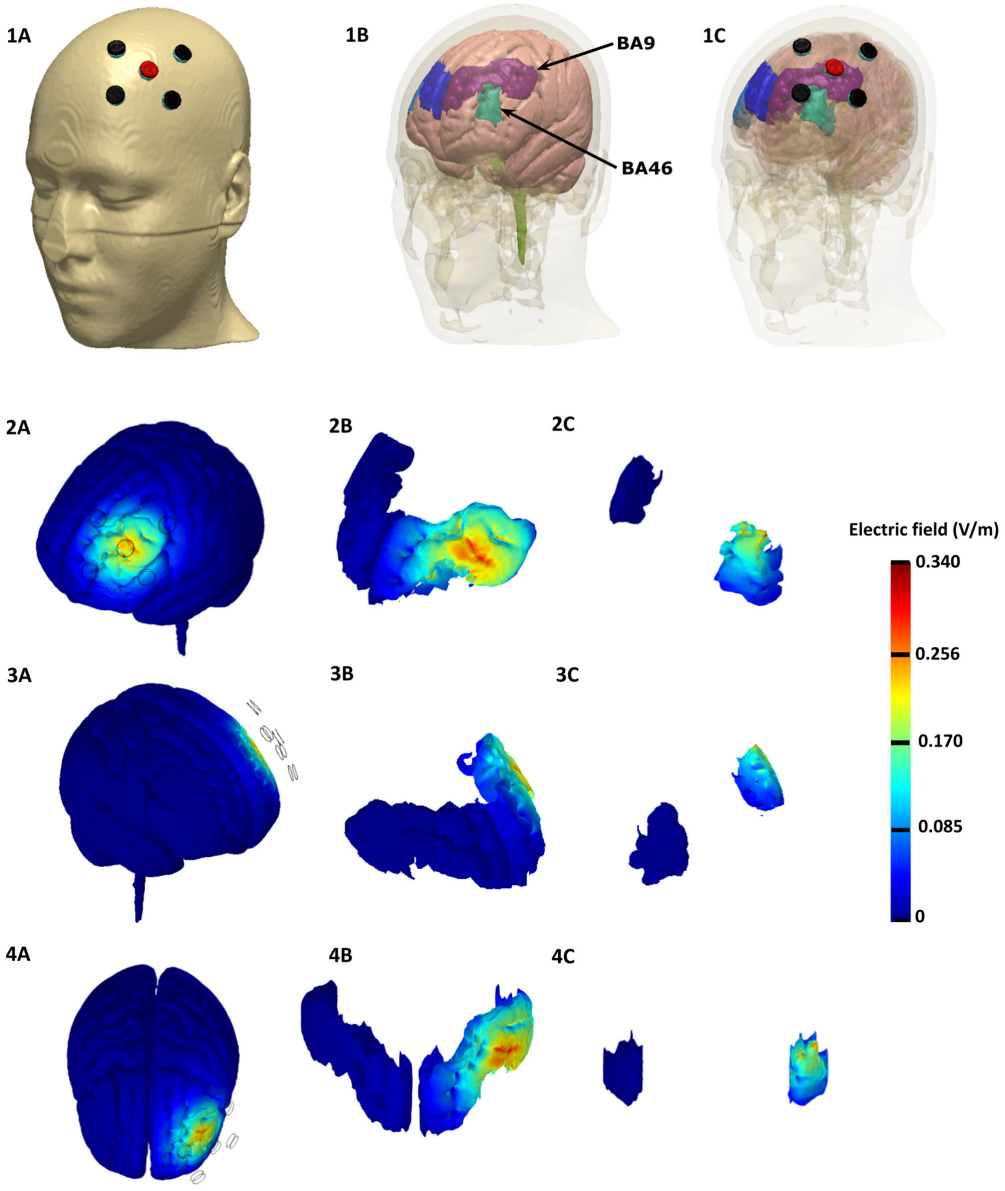
Finite element Model of the HD-tDCS Montage based on the representative MNI 152 template, Top row: Top row: Electrode Montage considered with anode depicted in red and the cathodes depicted in black (left). The underlying brain tissue mask with the individual Brodmann areas regions is depicted in the middle: left BA 9 (purple), left BA 46 (light green), right BA 9 (blue), and right BA 46 (light blue). The HD-tDCS montage in relation to the underlying gray matter and BA masks are shown on the right. Rows 2 − 4: Induced electric field magnitude plots (left side, right side, and top views). We plotted the whole brain in the first column, BA 9 in the middle, and BA 46 in the right.

The Laplace equation was solved, and the current density corresponding to 2 mA total current was applied. The induced surface electric field electric field (EF) magnitude was determined for the brain and Brodmann regions (BA 9 and 46, explored in Figure 2) separately. The HD montage was characterized by focal or restricted current flow to the region defined by the centered anode electrode extending to the outer cathode electrodes. TwomA current injection resulted in the EF peak value of 0.34*V*/*m* located directly underneath the active anode electrode. This peak region corresponding to the left BA-9 region overlapping the region underneath the F3 electrode induces current flow. The induced EF peak drops to 0.19*V*/*m* in the left BA 46 region. Minimal current flow in regions other than the left frontal cortex was noticed.

### 3.6. Electrophysiology Section of the Experiment

#### 3.6.1. High-Density Electroencephalography

**Figure 3:**
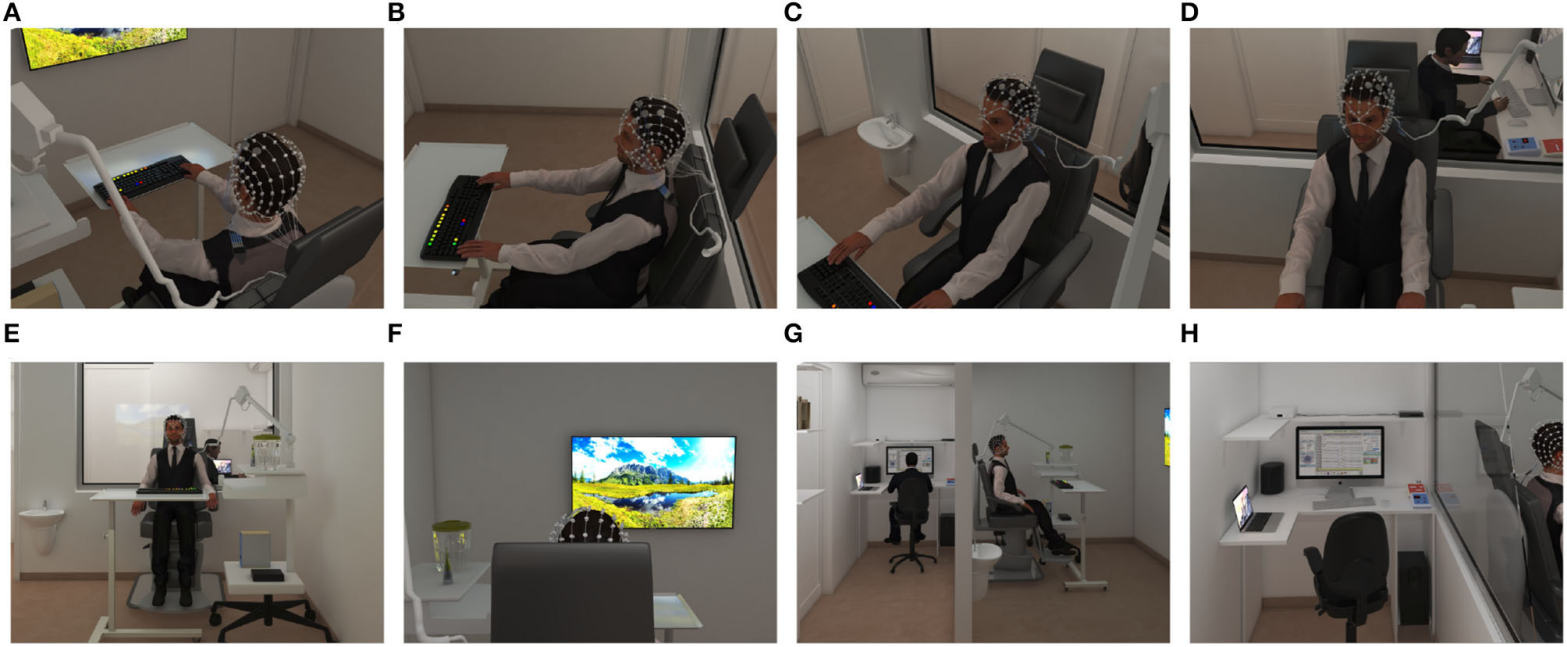
Clinical space and setup 3-Dimensional simulation for educational approach Exam Room; selected rendered-picture from Panoramic View with Camera at 200 cm Attitude: **(A)** 0° degree angle **(B)** 30° degree angle **(C)** 60° degree angle **(D)** 90° degree angle. **(E)** The perspective of the exam room was showed from the LCD's top. Control Room; selected rendered-picture from Panoramic View with Camera at 200 cm Attitude: **(F)** Exam room view was simulated from the clinician's perspective. **(G)** The perspective of both exam and control rooms. **(H)** Overview of the control room and setup.

The EEG signal accrued for 45 min per session using an EGI-Net (256-channel HydroCel Geodesic Sensor Net) and covered the whole head with less than 20 mm inter-electrode distances (**Figure 5A**). The 10 − 10 EEG Montage was applied with a reference on Vertex (Cz), recording 1,000 samples per second in a 22-bit analog-to-digital converter (Gajos and Wójcik, 2016) using NetStation 5.4.2 software (EGI, Eugene, OR, USA). Clinical setup was illustrated as a 3-Dimensional simulation in Figure 3.

#### 3.6.2. Universal Marker Interface

A Stimtracker (Cedrus Corporation, San Pedro, CA, USA) was employed to collect and register onset and end time points of different events, including picture presentation, TLQ, tDCS, and Stroop words on EEG signals. Additionally, responses to the Stroop task and TLQ self-assessment were collected via a mechanical keyboard (color programmable, SteelSeries, Apex-M800). Thus, we could analyze event-related potential (ERP) in the Time-Frequency domains. Clinical setup was depicted as a 3-Dimensional simulation in Figure 3H.

#### 3.6.3. Tinnitus Loudness Questionnaire (TLQ-Scale)

During sessions at the beginning and at the end of protocol blocks, patients responded twenty-one times to the TLQ “Scale your tinnitus loudness from 1*to*10.” Responses were collected by corresponding keys (*F*1 − *F*10) and longitudinally analyzed to provide a reliable scale (Breit et al., 2004) to monitor variations in perceived tinnitus loudness in response to the HD-tDCS and to present neutral or emotionally-valenced pictures. Correction of tinnitus loudness misperception modifies TLQ supported the surrogate endpoint for investigations on tDCS- dose efficacy. The dose was calculated after considering fixed-dose parameters and the TLQ response timestamp from tDCS stimulation beginning dose was calculated as per the given Equation 2.


(2) $$\begin{array}{l} \text{Dose}=\text{Intensity}\times \text{Timestamp of TLQ}. \end{array}$$

Ultimately, TLQ keeps the patients consciously attended to their tinnitus sound and helps ascertain evaluative conditional learning.

#### 3.6.4. Positive Emotion Induction

**Figure 4:**
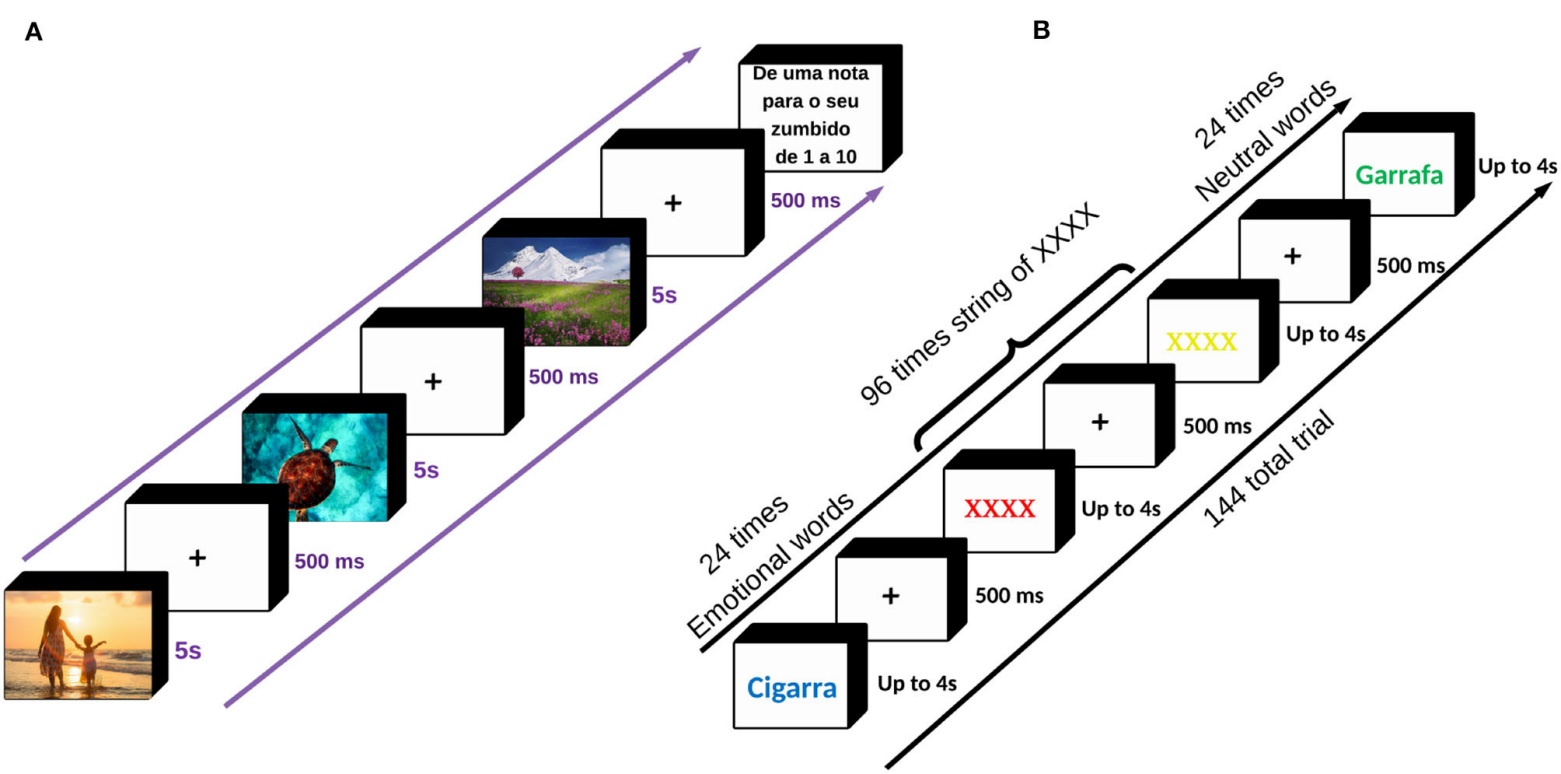
**(A)** The sequence of picture presentation. During the experiment, 80 neutral pictures and two-hundred positive pictures were displayed. Each picture was presented for 5 *s* with a cue of 500 *ms* in between. The positive emotion induction was concurrent with anodal stimulation, sham stimulation, or without any stimulation. Throughout the picture presentation, every 2 min we asked TLQ “Scale your tinnitus loudness from 1*to*10,” to keep the patients consciously attended to the perception of their tinnitus. The total time duration of the picture presentation was almost 28 *min*. **(B)** The sequence of trials in EST. In this task, six neutral and six emotional words, as well as the XXXX string (coming for two consecutive times after each word presentation), were randomly colored in red, blue, yellow, and green producing a total of 24 times for neutral and 24 times for emotional words as well as 96 times the XXXX string presentation, collectively 144 trials. The Patients were instructed to ignore the meaning of the stimuli and just respond to the colors seen by pressing one of the four corresponding keys: **Z, X, N, M** representing green, yellow, blue, and red, respectively, already programmed and colored on a LED keyboard. Before starting the task, the patients received a 10-s instruction explaining how to adjust their fingers over the four corresponding keys and how to act during the task. Either of the words or the XXXX string was presented for up to 4 s with a cue of 500*ms* in between, lasting 10*min* in total. EST was taken twice before and after the main intervention. List of words used in the Emotional Stroop Task. The patients were asked to list-out a set of words describing their tinnitus sound and rate either of the words from 1 (least annoying) to 5 (most annoying) to show how annoying it is. Six words with the highest frequency and annoyance were selected and matched with six neutral words in terms of syllables, word length, and frequency of use within the Portuguese language. ***“Neutral Wordlist”:*** {Garrafa, Banheiro, Luz, Antigo, Milho, and Patio}, and ***“Emotional Wordlist”:*** {Cigarra, Campainha, Tom, Apito, Grilo, and Radio} Source: Adapted from the Andersson et al. (2000; 2005).

Based upon the ECL mechanism, tinnitus neutral sound (conditional stimulus) can obtain negative valence after frequent pairing with negatively-valenced stimuli (unconditional stimulus) (De Houwer et al., 2001). Similarly, we hypothesized that the valence of tinnitus perception paired with positively- valenced stimuli might change to a less negative perception. In practice, specific emotional states can be induced by appropriate and controlled stimuli such as picture, sound, film, text, and virtual reality (Marchewka et al., 2014; Riegel et al., 2016). One of the most commonly applied and accepted stimuli for emotion induction is the use of pictures (Uhrig et al., 2016). The Nencki Affective Picture System (NAPS) is a database of standardized pictures for studying emotion and attention. It provides a detailed list of normative ratings in three dimensions of valence, arousal, and dominance elicited by each picture. It enables researchers to select stimuli triggering a specific customized range of emotions for their experiments (Marchewka et al., 2014). In the rating of the NAPS dataset, valence points to the positive vs. negative emotional state, whereas arousal points to the strength of emotional arousal or excitement (Citron et al., 2014). The pictures were rated using a modified 9-point Likert scale of Self-Assessment Manikin scale for arousal-ratio (Ar): 1= unaroused/calm, 9= aroused/excited; for Valence-ratio (Vr): 1= unhappy/annoyed, 9= happy/satisfied (Riegel et al., 2017). We employed a set of validated positive emotion-inducing pictures from the NAPS dataset to induce positive emotion simultaneously with CAAP of tinnitus to reduce the tinnitus negative valence. Pictures were aligned at a fixed location at the center of the screen in 1, 600 × 1, 200 pixels. We presented neutral pictures (4 < Vr <6 and Ar <6) included in Resting-state (rs) blocks and positive pictures (Vr> 6) included in PEI blocks developed in Superlab Software. The blocks contained 20 pictures, each one presented for 5*s*, followed by a 500 ms cue (+). Every single block ended with TLQ. The total duration of each block presentation was two min (Figure 4A).

**Figure 5:**
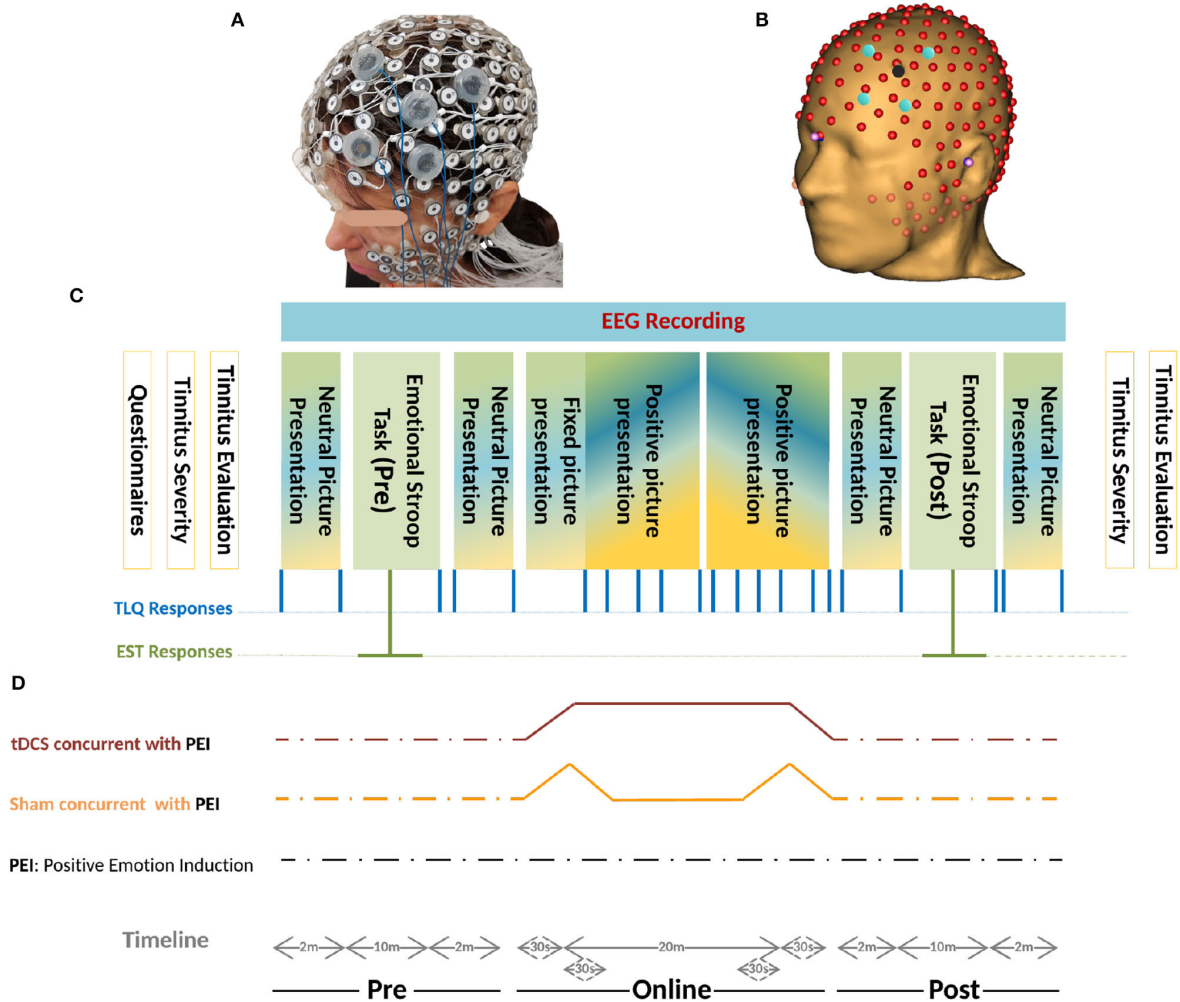
**(A)** Realistic Monatge for 256-channel HydroCel Geodesic Sensor Net, and The left-dlPFC as the Region Of Interest (ROI) is also mounted in the EGI-NET. **(B)** Co-registered 256-channel EEG, and HD-tDCS Montage on left-dlPFC based on the MR-driven head model. The stimulation electrodes located in electrode holders were placed near the EEG channels. The electrode holder for the anode is shown in red and that for the cathode is shown in blue. The electrode holders are filled with conductive gel. **(C)** Schemas of the protocol. Initially, the patients filled in different questionnaires- THI, TBF-12, TS, MDI, STAI-S6, and MSQ. Before and after the experiment, psychoacoustic parameters of tinnitus together with the Tinnitus Severity questionnaire were recruited. During the experiment, two sets of pictures were presented with different Valence-ratio (Vr) and arousal-ratio (Ar) rates selected from the NAPS dataset. Neutral pictures (4 <Vr< 6 and Ar< 6) were included in four rs blocks and the positive pictures (Vr> 6) in 2 × 5 consecutive PEI blocks. Each PEI block initiates with four pictures (Vr> 6 and Ar>6) followed by sixteen pictures (Vr> 6) randomly selected with no replacement from a one-hundred-positive-picture set (Figure 4A). Generally, every single block contained 20 pictures each presented for 5*s* followed by a cue (+) of 500*ms*. The blocks were randomized between sessions and between patients but presented in the same order within-session. The total duration of each block presentation was two min. The TLQ “Scale your tinnitus loudness from 1 − 10” was displayed 21 times, and presented in the following order: before-after each resting-state block (containing neutral pictures) and PEI block (containing positive pictures), and after each Emotional Stroop task. Throughout the experiment, the responses of TLQ and EST in blue and green lines, respectively, as well as EEG signals were all recorded via Superlab software (Cedrus Corporation, San Pedro, CA, USA). The total duration of the experiment ranged between 40 and 45 min with regard to the reaction time of patients in EST and responding to TLQ. **(D)** Timeline of all three sessions of study including anodal stimulation-picture presentation, sham stimulation-picture presentation, only picture presentation is illustrated. In all three sessions, the sequence of presenting pictures, TLQ, and EST are the same as those described in part C. Electrical stimulation periods (sham/Active) were illustrated in movies of Supplementary Materials.

The rs blocks were constructed with neutral pictures that were randomized between sessions and between patients but were presented in the same within-session order. Neutral pictures were selected for evaluating baseline neural activity, which is not elicited by a task. So, the rs block was displayed four times to provide a reference and cover all possible repeated measures of rs brain activity that might be affected by previous tasks (Figure 5C).

#### 3.6.5. Justification for Resting-State Blocks and Using EEG

EEG is employed to assess the changes in neural activity (Coffman, 2014) before, during, and after administration of the tDCS (Schmidt et al., 2014; Labruna et al., 2016). tDCS can modify EEG components and brain activity that denote neuro-cognitive responses to stimuli. A more in-depth comparison is necessary for understanding the relationship between tDCS effects, changes in EEGs, and related changes in cognition (Bikson et al., 2018). A 20-min positive picture presentation, fully randomized between patients and sessions was designed in ten-blocks constructed from 5-consecutive PEI blocks repeated twice. This was done because positive picture repetition could not modulate Late Positive Potential (Mastria et al., 2017). Each block initiated with four pictures (Vr> 6 and Ar> 6) followed by sixteen pictures (Vr> 6) randomly selected with no replacement from a one-hundred-positive-picture set (Figure 5C).

According to the Arousal-Biased Competition theory, arousal enhances emotional stimuli processing but impairs neutral stimuli (Mather and Sutherland, 2011; Lee et al., 2012, 2014; Sutherland and Mather, 2012; Singh and Sunny, 2017). Since this theory supports biased salient processing, we placed high-arousal-valence pictures (HAV) pictures before high-valence Pictures (HV) pictures per PEI block.

### 3.7. Emotional Stroop Task

Negative appraisals about tinnitus and frequent coincidence of tinnitus perception with negatively-valenced stimuli can reinforce the corresponding negative cognitive-emotional value and can lead to attentional bias. Through this process, tinnitus sound could be prioritized for further processing and other competing stimuli could be suppressed (Ghodratitoostani et al., 2016b). EST is a well-established paradigm to examine attentional bias and interference effects of emotional stimuli on cognitive processing (Williams et al., 1996; Dresler et al., 2009). In this task, a set of emotional words (relevant to tinnitus) and neutral words (irrelevant to tinnitus) were presented in different colors. The patients were instructed to respond to the color as quickly as possible while reading silently and ignoring the meaning of the words presented. Delayed response to the color of emotionally-laden words vs. neutral words indicates the emotional interference effect and attentional bias to valenced information (Williams et al., 1988, 1996; Andersson et al., 2005). In the current study protocol, the stimuli of EST were provided through the following procedures:

1. Patients were asked to list words that describe their tinnitus sound.
2. Patients rated each word according to its annoyance intensity from 1−5 (the least annoying words were rated 1 and the most annoying as five).
3. A final list of six tinnitus descriptors as the most annoying and frequently reported was chosen.
4. Six neutral words were matched in terms of syllables, word length, and frequency of use within the Portuguese language
5. Six matched-character XXXX-strings were used to identify the semantic effect.

Whole lists (emotion, neutral, and XXXX-string) were randomly assigned to red, blue, yellow, and green colors for the construction of the EST protocol (Andersson et al., 2000, 2005). Before EST, instructions were displayed for 10*s* giving information about the test and how the patients should adjust their fingers over four keys: **Z, X, N, M** for green, yellow, blue, and red, respectively already programmed and colored on a LED keyboard. Either of the stimuli was then presented for up to 4*s*, followed by a 500*ms* cue (+). Each of the neutral and emotional words was randomly presented four times (N=48 trials) followed by two consecutive XXXX-string (N = 96 trials) to avoid the carryover effect of the emotional words (see Figure 4B). In this study, EST with 144-trials was obtained twice (once before and once after intervention) in each session. Superlab Software (Cedrus Corporation, San Pedro, CA, USA) was used to collect and document the responses together with the corresponding reaction times.

### 3.8. Magnetic Resonance Imaging (Structural and Functional)

Resting-state functional Magnetic Resonance Imaging (fMRI) applied on recruited patients who underwent a 3.0*T* MRI examination (Achieva 3.0T X-series, Philips Medical Systems, Best, The Netherlands) using a 32 channel sense head coil at HCRP-FMRP-USP, Brazil. The Ethics Committee of the institution approved the study, and prior-written informed consent from all patients was obtained. Data were collected from September to December 2017, With a minimum of 48 h after their active session. Before each imaging session, the patients responded to the same battery of questionnaires taken throughout the main experimental sessions. During scanning, the patients were asked to raise their thumb if they still perceived the tinnitus sound. To do so, we assured them that the MRI scanner noise would not mask tinnitus sound. Below are the characteristics of the imaging procedures.

- **Structural imaging**
  – **3DT1:** The sequence was acquired in the sagittal plane with a1 mm isotropic voxel 3D T1-weighted MPRAGE sequence. Phase and magnitude data were stored. The sequence parameters: 3.2/7.0/8 (TE/TR/Flip angle); slice thickness = 1mm and matrix = 240 × 240, allowing isotropic voxel of 1.0 × 1.0 × 1.0mm, the field of view (FOV), 240(*FH*) × 240(*AP*) × 170(*RL*) mm^3^, SENSE, 2.
  – **3D volumetric Fluid-Attenuated Inversion Recovery sequence (FLAIR):** The sequence was acquired in the Sagital plane. The sequence parameters were: 343/5, 000/1, 600 ms (TE/TR/TI); spatial resolution, 1.0 × 1.0 × 1.0 mm^3^, the field of view (FOV), 240(*FH*) × 240(*AP*) × 180(*RL*) mm^3^, SENSE,2.
  – **Diffusion Tensor Imaging (DTI):** The sequence acquired in the AXIAL plane using a Fast Spin-Echo echo-planar imaging (EPI) in 32 orthogonal directions. Sequence parameters: 65/8.888/90 (TE/TR/Flip angle); spatial resolution voxel of 2 × 2 × 2 mm, the field of view (FOV) 256, acquisition matrix of 128 × 128, SENSE,2.
- **Functional Imaging**
  – **Resting-state (rs):** 200 volumes, 29 slices in ascending order without gaps, 4-mm slice thickness, voxel size = 3 × 3 mm, field of view = 240 × 240 mm, TR/TE = 2, 000/30 ms. The silent sequence was designed by setting to maximal (level 5) “soft-tone” parameter offered by the MRI equipment, which reduces the gradient slew rate, leading to lower coil vibration levels (Rondinoni et al., 2013).

The resting-state fMRI reveals correlated activity between mostly separate but functionally connected brain regions and explains the integrity of functional brain circuits in tinnitus patients (Maudoux et al., 2012). The present study aimed at testing whether fMRI "rs" connectivity patterns and dynamics in auditory and emotional networks differ between tinnitus patients and healthy controls. This might offer a better insight into the neural basis underlying the tinnitus pathophysiology and suggests more effective and customized treatment options.

## 4. Statistical Study Design and Sample Size Calculation

Food and Drug Administration (FDA) [21 CFR 314.126] recommends performing a well-controlled study for a new drug approval that characterizes with:

- A transparent statement of the research objectives
- A study design allows a valid comparison with control to provide a quantitative assessment of drug effect. placebo-like, Dose- comparison, No treatment, and Active and Historical control.
- Accurate recruiting and inclusion protocol to guarantee selected subjects have the desired condition.
- Reliable randomization procedure to assign recruited subjects with a minimum bias to control and treatment groups.
- Sufficient sample size and Blindness arrangements to minimize the bias on subjects, observers, and data analysts.
- Robust and reproducible assessment of measuring factors.
- Adequate credibility, power, and confidence to evaluate the drug efficacy.

**Figure 6:**
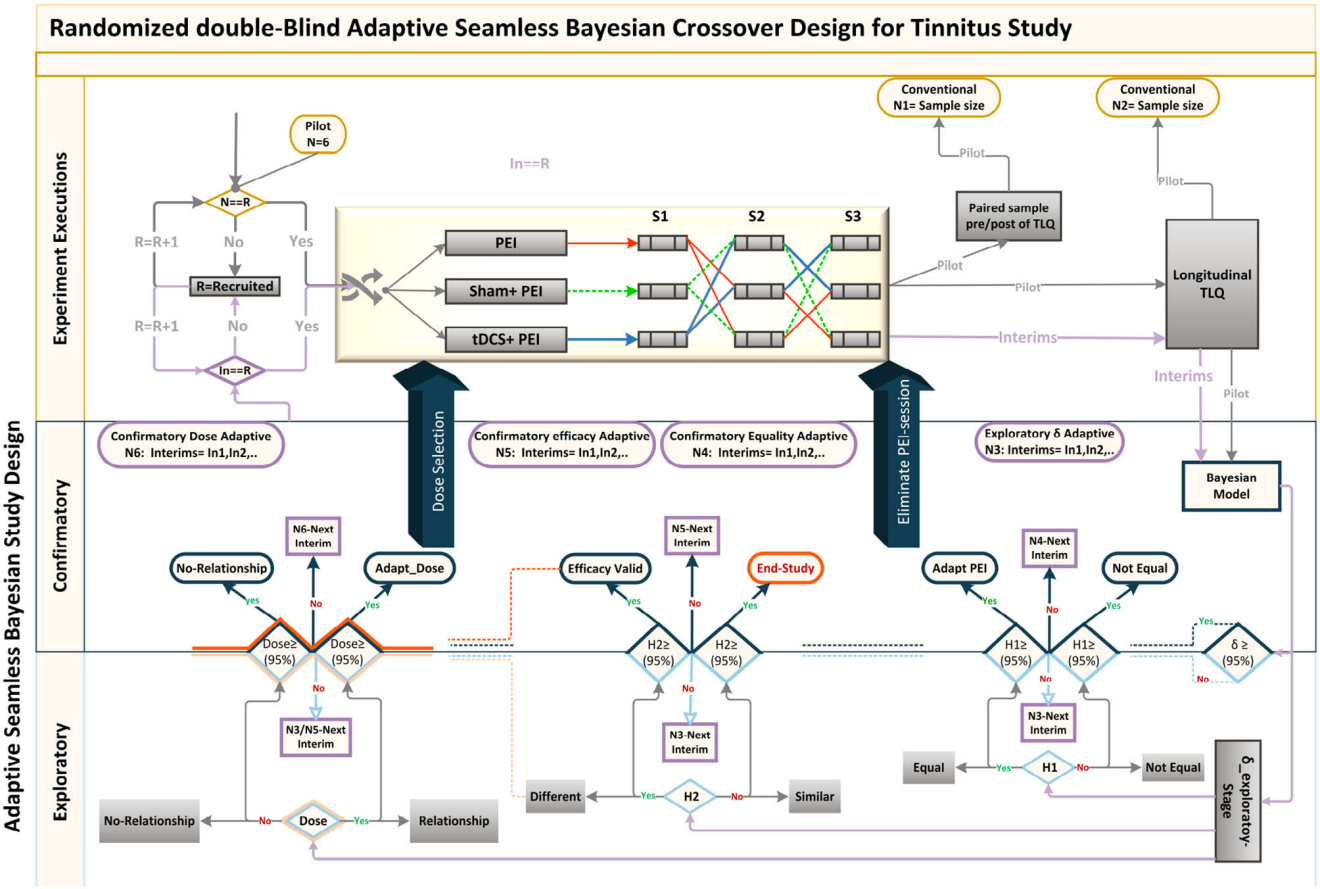
Adaptive Seamless Bayesian Study Design: Experiment execution: Study recruitment, randomization, interim applications, three crossover interventions were illustrated. Interventions were PEI, “tDCS + PEI,” and “Sham + PEI.” Six patients were recruited and randomly assigned to the interventions based on the William-design. Conventionally, either (paired sample analysis)TLQ difference between before and after interventions or (longitudinal analysis) repeated measures of surrogate endpoint TLQat specific time-points could be used for sample size calculation. Frequent interim analyses based on Markov-chain Monte Carlo estimate Bayesian posterior probability distributions, with multiple imputation and estimation of unknown trial parameters and patient outcomes. Exploratory and confirmatory stages: The seamless design connects independent trials inside a single study. An Adaptive-Seamless Bayesian (ASB) design consolidates with two stages exploratory-stage and confirmatory-stage. The Minimum Clinical Efficacy (δ) is defined as 50% of the observed effectiveness for PEI treatment, which was determined after assessing the variability observed during the online period. Since PEI has not yet been practiced for the tinnitus population, the Bayesian model utilized the probabilistic model to represent all uncertainties within the model (Gelman et al., 2013), both the hypotheses and prior knowledge (Processed-δ or non-credible δ). Testing hypotheses are **H**_**1**_: The conscious pairing of PEI simultaneously with tinnitus perception results in the correction of loudness misperception. In the current ASB the following hypotheses were tested simultaneously, **H**_**2**_: Active HD-tDCS on the left-DLPFC but not sham facilitates correction of the loudness misperception, and **Dose**: A dose-response relationship between HD-tDCS dosage and correction of loudness misperception exists. Since the processed-δ is not changed, interim analyses on hypotheses in exploratory and confirmatory stages will be processed. The results of the hypotheses are partially valid within the uncertainty range of the processed-δ. When δ becoming valid decision making and adaptation on *H*_1_ and *H*_2_ is possible. However, as soon as Processed-δ is changed, the hypotheses needed to be reevaluated. When δ and *H*_2_ became credible concurrently, regulating the dose and judgment about the dose-response relationship is possible. In other cases, the validity of dose-related parameters depends on processed-δ and efficacy uncertainty.

Analogously with well-controlled study characteristics,TLQ was collected through paper-based Tinnitus Severity Questionnaires obtained before and after the experiment, and the pressing corresponding keys on the keyboard during the experiment. TLQ as the surrogate endpoint was measured to test the superiority of concurrent positive emotion induction (PEI) and HD-tDCS against only PEI as an active control. Minimum Clinical Efficacy (δ) is indicated to test superiority. An inappropriate selection of δ can influence sample size calculation and inference validity. The FDA suggests selecting δ between 25 and 50% of the effect size of the active control (Chow et al., 2017). Since PEI has not yet been practiced for the tinnitus population, a Bayesian crossover adaptive “seamless trial” (Chow and Tu, 2008) was designed to study δ and dose selection. The Bayesian model employed the probabilistic model to represent all uncertainties within the model (Gelman et al., 2013), both the hypotheses and the prior knowledge δ. For this, we adapted the sample size for the subsequent confirmatory trial by measuring surrogate endpoints and single session HD-tDCS_4×1_ dose-response relationship (Figure 6).

The seamless design combines two independent trials inside a single study. A seamless adaptive design most often incorporates two-stages before adaptation (exploratory-stage) and after adaptation (confirmatory-stage). The exploratory stage aims at obtaining information regarding the uncertainty of the testing treatment to apply adaptation. It may be similar to opening the door for investigators to stop the trial beforehand due to single or multiple issues based on safety, futility, and accrued data efficacy. The scope of the confirmatory-stage is to verify preliminary findings from the exploratory-stage. The major advantage of the two-stage adaptive seamless study is in its capability of merging collected data from both stages to derive a more accurate and reliable inference. Six patients were recruited to conduct the current clinical pilot study to calculate the sample size and to investigate the following hypotheses:

- The conscious pairing of PEI simultaneously with tinnitus perception results in the correction of loudness misperception
- Active HD-tDCS on the left-DLPFC but not sham facilitates correction of the loudness misperception.
- A dose-response relationship between HD-tDCS dosage and correction of loudness misperception exists.

### 4.1. Study Roadmap

We propose an innovative Adaptive-Seamless Bayesian (ASB) method that can revolutionize improved efficacy in clinical trial ethics, design, execution, and performance. ASB assists the investigators in controlling unknown potential confounders (i.e., unforeseen issues at the beginning of the study) considering any prior knowledge. The adaptation can be individually or collectively employed for measurement factors, sample size, randomization, clinical endpoints, biomarkers, and surrogate endpoints to achieve significant credibility and reproducibility in ongoing studies. Consequently, testing hypotheses across the ASB method results in well-controlled, accurate, and time-cost efficient studies. To our knowledge, this is the first-ever crossover study with active control (PEI) applied for the tinnitus population. Conventional approaches in study design need to conduct at least three dedicated consecutive studies to investigate the hypotheses mentioned above. First, to conduct a prospective comparative research study to learn and quantize the effect of positive emotional induction (with validated images) in the tinnitus population. Subsequently, a comparative crossover or parallel study could be performed to investigate the superiority effect of “tDCS concurrent with PEI” but not “Sham concurrent with PEI” on the correction of loudness misperception and tinnitus bothersome against the PEI. Overall, to select the effective dose, a dose-response study must be run to establish the single-session response relationship to the applied doses. Understanding the minimum and maximum effective doses lead us to adequate and controllable doses for multisession treatments. ASB method enables the coverage of all hypotheses collectively within an adaptable progressive study. The sample size should be adapted to all progressive stages of the study toward maximizing the credibility margin (95%) in testing hypotheses. Our pilot study results demonstrated that the coefficient of variance showed statistically significant differences (90% power at 1% significance level) between the loudness perception of tinnitus during PEI and neutral picture presentations creating prior knowledge of the δ exploratory stage. Based on prior knowledge, interims (sample size *N*_3_) of the confirmatory stage are computed to derive δ value within the desired credibility margin. In every single interim of δ confirmatory (*N*_3_), the posterior delta is calculated and proceeds in one of the following states to generate the processed-δ:

1. Regardless of the difference between posterior and anterior δ (meaningful or not), when δ is not placed in the desired credibility margin, the anterior δ remains acceptable. This occurs due to the magnitude of certainty, and upcoming recruitment is performed by the next interim of the δ validation sample size in the confirmatory stage.
2. The difference between posterior and anterior δ is significant and placed in the desired credibility margin. Therefore, the anterior δ replaces the posterior, and updated δ shapes new prior knowledge for the δ exploratory stage. This stage adapts all credibility margins of other hypotheses and corresponding sample sizes. However, upcoming recruitment builds upon the adapted sample size interims of the new δ within the confirmatory stage.
3. Though insignificant, the difference between posterior and anterior δ is placed in the desired credibility margin. Therefore, the anterior δ is valid, and the δ confirmatory stage ends. Upcoming recruitments are then used by the corresponding interims of the hypothesized sample size in the confirmatory stage. Eventually, in every interim after δ confirmation, “valid δ” applies to test hypotheses with the corresponding sample size interims.

During each interim analysis, the corresponding processed-δ applies to test the hypotheses. Possible results for testing hypotheses are enumerated below:

- ***H***_**1**_: Reduction in tinnitus loudness perception during “Sham concurrent with PEI” is similar to only PEI. When δ is valid, evidence in favor of *H*_1_ in a credible margin leads to the conclusion that “SP” is similar to PEI, so the study adapts with the PEI session elimination. In contrast, when the evidence is against *H*_1_ in a credible margin, “SP” differs from the PEI. When δ is valid and the *H*_1_ result is partially acceptable, it supports temporary decision making in an ongoing study. Therefore, the study recruitment proceeds with *N*_4_ interims. When processed-δ is present, regardless of its credibility, the certainty ratio is involved in the *H*_1_ result. Therefore, the study recruitment goes on with the *N*_3_ interims.
- ***H***_**2**_: Reduction in tinnitus loudness perception during tDCS concurrent with PEI “tP” differs from that in PEI. When δ is valid, evidence in favor of *H*_2_ in a credible margin leads to the conclusion that automatic content recognition short “tP” does not differ from only PEI. However, if the evidence is against *H*_2_ in a credible margin, it leads to the conclusion that “tP” is not different from only PEI, so the study ends. When δ is valid and *H*_2_ is partially acceptable, temporary decision making in an ongoing study can be corroborated. The study recruitment proceeds with *N*_4_ interims. While processed-δ is present, regardless of its credibility, the certainty ratio is involved in the *H*_2_ result, so the study recruitment continues with *N*_3_ interims. The results of *H*_2_ drive the dose-response relationship investigation with partial certainty, except the rejecting state of *H*_2_ within a credible margin.
- **Dose:** A dose-response relationship between HD-tDCS dosage and correction of loudness misperception exists. Valid δ with credible *H*_2_ leads to either of the following conclusions:
  - If Dose is accepted in a credible margin, it establishes the dose-response relationship and the minimum and maximum dose can be defined. The study adapts to the subsequent multisession clinical study.
    The evidence against Dose in a credible margin shows no dose-response relationship. Valid δ together with non-credible *H*_2_ results provides partially acceptable results for Dose. This supports temporary decision making in the ongoing study, and the study recruitment moves on with N5 interims. Valid δ, together with the credible *H*_2_ result, provides partially acceptable results for the Dose that supports temporary decision making in the ongoing study and the study recruitment moves on with N6 interims. When processed-δ is present, the Dose result is available with certainty ratio regardless of *H*_2_ results credibility. Therefore, the study recruitment continues with *N*_3_ interims.

### 4.2. Data Description

**Figure 7:**
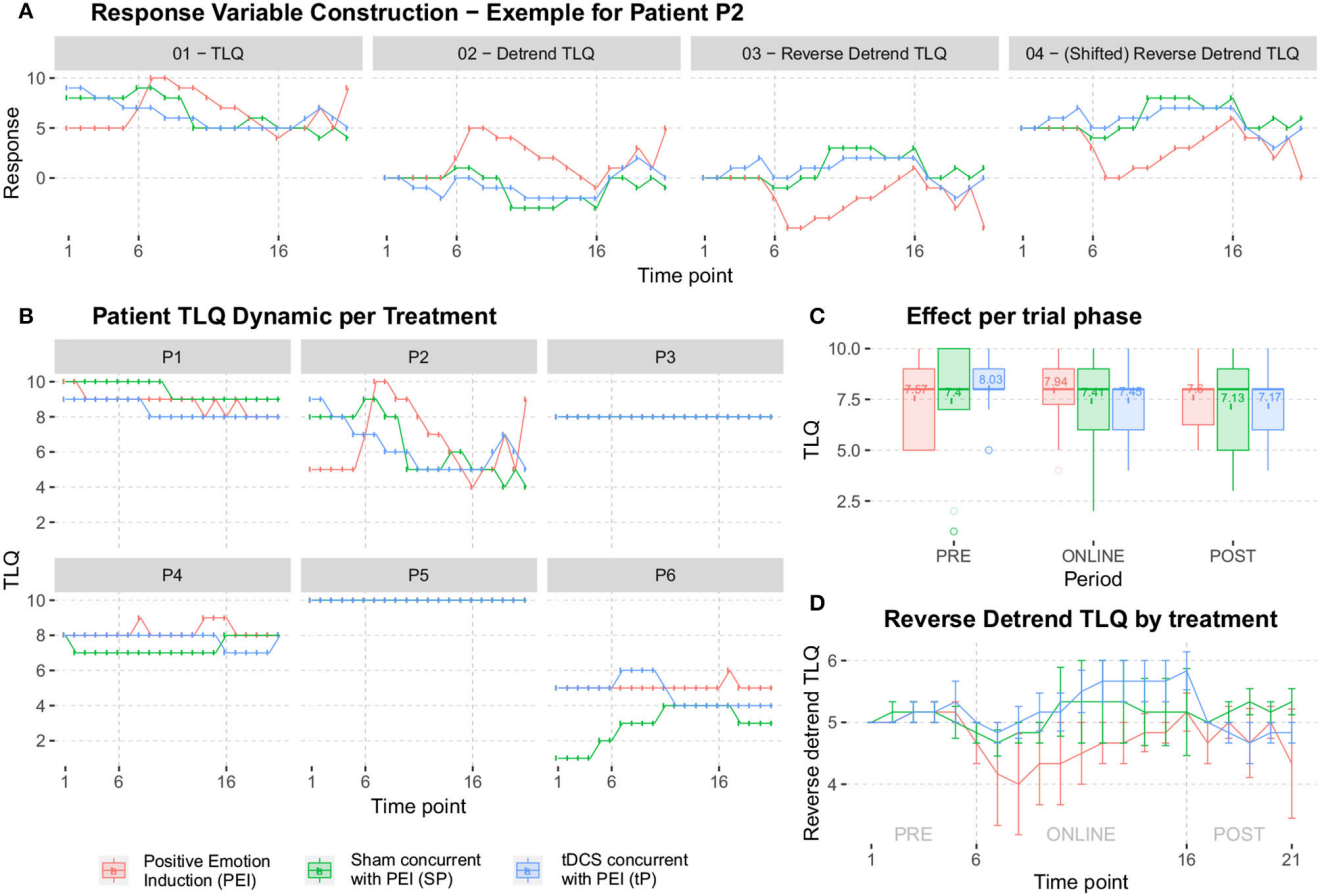
**(A)** The construction of the response variable was analyzed later. In (a-1) it starts from the response collected in the pilot study (TLQ); In (a-2) the tendency is isolated by extracting the stability point in each segment PRE-ONLINE-POST (obtaining the Detrend TLQ response); In (a-3) reverse the Detrend TLQ response to allow a direct interpretation between “the more, the better” and “the less, the worse” (obtaining the Reverse Detrend TLQresponse); In (a-4) we move the Reverse Detrend TLQ response to the positive axis for mathematical convenience (obtaining the Shifted Reverse Detrend TLQ response, which is equivalent to the previous one). **(B)** All individual participants in the pilot study. Two patients reported constant response TLQ during all sessions. **(C)** The box-plots summarize the range of the responses, which highlight the median of treatment responses given periods. **(D)** The average of all patients, added by one standard deviation.

Six subjects recruited for this study underwent a 3-session crossover trial to create Bayesian inferences to calculate sufficient sample size for efficacy, safety, and single session dose-response relationship. The studied relationship corresponds to the behavior of a transformation performed on TLQ, "surrogate endpoint," (Figure 7A). During the trial, TLQ was recruited longitudinally at 21 different time points as well as before and after each session through questionnaires. TLQ is a 10-point Likert scale that represents patients' tinnitus loudness perception (Figure 7B). Two patients reported no change in their TLQ scores through all the recorded time points. Accordingly, longitudinal TLQ was segmented into Pre-, Online-, and Post-TLQ where box-plots of the segmented measures are illustrated in Figure 7C. The maximum variation was observed in the “SP” session. Nevertheless, the PEI behavior showed similarity to that of “SP” considering its inception box-plots. Moreover, the presentation of “tP” offered visual evidence toward reducing post-TLQ vs. Pre-TLQ. Initially, longitudinal TLQ (Figure 7a-1) responses were scaled to obtain Detrend TLQ response (Figure 7a-2). We considered the difference between a particular timepoint from the starting point (reference) of the corresponding segments for reductions in the confounding effects of longitudinal carry-over. We aimed to assess the cumulative variation at each timepoint against the reference of the corresponding segment. Correspondingly, the dataset was preprocessed considering the last time point of pre segment “*T*_1_ to *T*_5_” as a reference of the online segment “*T*_6_ to *T*_16_” timepoints. The Online segment's last time point was considered the reference of post segments “*T*_17_ to *T*_21_.” It can be empirically confirmed that during segment-pre neutral-picture presentation along with the EST revealed a minor change in corresponding TLQ time point responses. In Figure 7D Reverse Detrended TLQ responses were presented considering an average of the response of all patients at each time point by adding one standard deviation above and below. We considered the reverse response reduction with a view to better understand the reduction in the loudness perception. The differences were exhibited positively. On considering the online segment, all treatments demonstrated reductions in TLQ (i.e. increase in reverse detrend TLQ). Moreover, only in the PEI session, a dramatic rise in the average of the TLQ responses at the time-point *T*_8_ was observed probably due to the small sample size of the pilot. We noticed the effect of the intervention through the segment-post gradually, but not linearly depreciated-except the “SP” sessions, which seems almost constant.

#### 4.2.1. Minimum Clinical Efficacy (δ)

**Figure 8:**
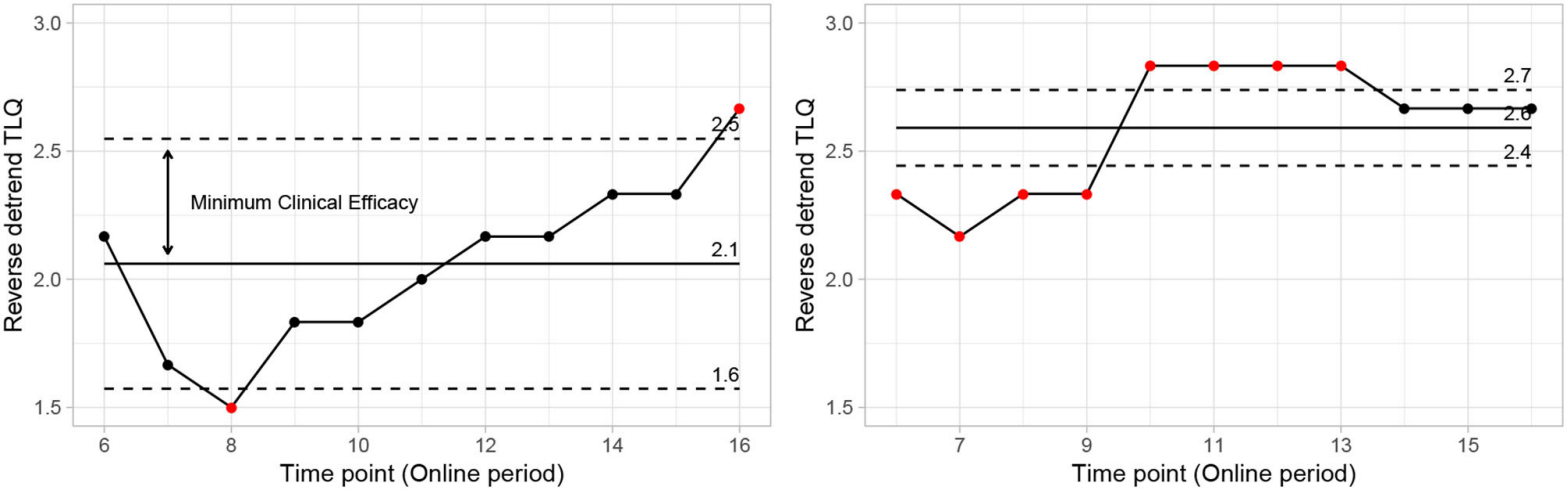
Control chart showing mean points and natural limits from data (PEI left side vs. “SP” right side). Points outside limits reflect fundamental changes in expected behavior. Reflecting significant changes in the average behavior of the reverse detrend TLQ. On the other hand, the variation around the mean and internal to the limit shows the usual behavior with small changes due to randomness.

The δ is defined as 50% of the observed effectiveness for PEI treatment, which was determined after an assessment of the variability observed during the online period, using comparative methodologies. It is noteworthy that the amount of δ forms part of the adaptive investigation in the methodology outlined here as depicted in the study design (Figure 6). δ was recalculated at each intermediate phase of the experiment. So, the precision applied on “processed-δ” has significant implications on intermediate sample size calculations. The descriptive δ obtained from the pilot study indicates that 50% of the variation observed in the PEI treatment corresponded to the displacement of approximately 0.4 units from the mean (Figure 8). Variation of less than 0.4 can be considered a clinically insignificant effect. Resultantly, each interim analysis required re-estimation of the δ and update of the active control PEI effect, leading to a credible interval adaptation. Subsequent interims are adapted for investigating the credibility of the new δ.

### 4.3. Bayesian Modeling for Single-Session Dose-Response Adaptive Seamless Study Design

We proposed the superiority effect of “tP” treatment vs. “SP” or PEI. Correspondingly, TLQ as the dependent variable was transformed into the reversed detrended version (Figure 7). Additionally, a theoretical model (that contemplates the behaviors observed in Figure 7D) was developed to evaluate the following hypotheses of interest. The hypotheses that tested with the Minimum Clinical Efficacy (δ) are:

- Test the equality and inferiority/superiority between the applied treatments. The hypotheses, where δ is the minimum clinical efficacy, to be tested are:
  $$\begin{array}{l} \begin{array}{c} H_{0}:\mu_{\text{“S}{\text{P}}^{\prime \prime }}-\mu_{\text{PEI}}=0\\ H_{A}:\mu_{\text{“S}{\text{P}}^{\prime \prime }}-\mu_{\text{PEI}}\neq 0 \end{array}\;\begin{array}{c} H_{0}:\mu_{\text{“t}{\text{P}}^{\prime \prime }}-\mu_{\text{PEI}}\leq \delta \\ H_{A}:\mu_{\text{“t}{\text{P}}^{\prime \prime }}-\mu_{\text{PEI}}>\delta \end{array} \end{array}$$
  $$\begin{array}{l} \;\begin{array}{c} H_{0}:\mu_{\text{“t}{\text{P}}^{\prime \prime }}-\mu_{\text{“S}{\text{P}}^{\prime \prime }}\leq \delta \\ H_{A}:\mu_{\text{“t}{\text{P}}^{\prime \prime }}-\mu_{\text{“S}{\text{P}}^{\prime \prime }}>\delta \end{array} \end{array}$$
- test the carry-over effect, by the hypothesis
  $$\begin{array}{l} H_{0}:\mu_{\text{PRE}}=\mu_{\text{POST}}\\ H_{A}:\mu_{\text{PRE}}\neq \mu_{\text{POST}} \end{array}$$
- Investigate the influence of self-perceived severity of tinnitus on the effect of treatment-experienced. This was done by adding to the model, as a predictor variable, the first response given by the patient, at *T*_1_, of the Pre period;
- Investigate the minimum clinical efficacy (as illustrated in Figure 8, on the left) using the response estimated by the model, in each interim analysis;
- Investigate the dose-response dynamics, populationally, and individually. This was done considering the fixed effects of independent variables in models for the interpretation of population dynamics together with the inclusion of random effects for the individualization of interpretations;
- Provide a guideline for an adaptive study design in calculating sample size. This was done using sequential sampling methodologies and also methodologies for sampling in adaptive studies.

Naturally, hierarchical modeling was conducted using the Bayesian approach (Migon et al., 2014) as an adaptive strategy in endpoint, sample size, and single session dose-response relationship. The formal description of the proposed statistical model is given described as follows.

#### 4.3.1. Statistical Model Formulation

We first consider the response variable *Y*_*ij*_, which represents the observed dynamics (increase or decrease) between the responses throughout 21-time points of each session associated with *i*-th individual in the *j*-th time point. A possible model for the understanding of the average dynamics behavior can be formulated as follows.


(3) $$\begin{array}{l} Y_{ij}|\gamma_{\text{Id}},\gamma_{\text{Od}},\mu_{ij},\sigma^{2}\overset{\text{ind}}{\sim }N\left(\mu_{ij},\sigma^{2}\right),\\ \;\gamma_{\text{Id}}|\Lambda_{\text{Id}}\overset{\text{iid}}{\sim }{\text{N}}_{9}\left(0,\Lambda_{\text{Id}}\right),\\ \;\gamma_{\text{Od}}|\lambda_{\text{Od}}^{2}\overset{\text{iid}}{\sim }\text{N}\left(0,\lambda_{\text{Od}}^{2}\right), \end{array}\begin{array}{c} i=1,\ldots ,6,\\ j=1,\ldots ,21, \end{array}\begin{array}{c} \text{(subjects)}\\ \text{(time points)} \end{array}$$

where **Λ**_Id_ is a diagonal matrix. Furthermore, in this context, the parameter μ_*ij*_ ssunctional relation


$$\begin{array}{l} \mu_{ij}=\{\begin{array}{cc} G_{\text{P}}\left(j,a_{i},b_{i}\right)+\gamma_{\text{Od}} & \text{if }j=1,\ldots ,5,\\ G_{\text{T}}\left(a_{i},f_{i}\right)+\gamma_{\text{Od}} & \text{if }j=6,\\ G_{\text{O}}\left(j,c_{i},d_{i},e_{i},f_{i}\right)+\gamma_{\text{Od}} & \text{if }j=7,\ldots ,16,\\ G_{\text{S}}\left(j,c_{i},g_{i},h_{i}\right)+\gamma_{\text{Od}} & \text{if }j=17,\ldots ,21, \end{array}\begin{array}{c} \text{(PRE period model)}\\ \text{(Transient period model)}\\ \text{(ONLINE period model)}\\ \text{(POST period model)} \end{array} \end{array}$$

with


$$\begin{array}{l} \begin{array}{c} G_{\text{P}}\left(j,a_{i},b_{i}\right)=a_{i}+b_{i}\left(j-5\right),\\ G_{\text{T}}\left(a_{i},f_{i}\right)=\frac{f_{i}+a_{i}}{2}+\gamma_{T\text{Id}},\\ G_{\text{O}}\left(j,c_{i},d_{i},e_{i},f_{i}\right)=f_{i}+\frac{c_{i}-f_{i}}{1+\exp\{-d_{i}\left(j-e_{i}\right)\}},\\ G_{\text{S}}\left(j,c_{i},g_{i},h_{i}\right)=c_{i}+\left(g_{i}-c_{i}\right)[1-\exp\left\{-h_{i}\left(j-16\right)\right\}], \end{array}\begin{array}{c} \text{(Linear growth/decay model)}\\ \text{(Transient point between PRE and ONLINE)}\\ \text{(Four parameters logistic growth/decay model)}\\ \text{(Exponential growth/decay model)} \end{array} \end{array}$$

where the quantities *a*_*i*_, *b*_*i*_, *c*_*i*_, *d*_*i*_, *e*_*i*_, *f*_*i*_, *g*_*i*_ and *h*_*i*_, are given by


$$\begin{array}{l} a_{i}=x_{ia}^{⊤}\beta_{a}+\gamma_{a\text{Id}}\;b_{i}=x_{ib}^{⊤}\beta_{b}+\gamma_{b\text{Id}}\;c_{i}=x_{ic}^{⊤}\beta_{c}+\gamma_{c\text{Id}}\;d_{i}=x_{id}^{⊤}\beta_{d}+\gamma_{d\text{Id}}\\ e_{i}=x_{ie}^{⊤}\beta_{e}+\gamma_{e\text{Id}}\;f_{i}=x_{if}^{⊤}\beta_{f}+\gamma_{f\text{Id}}\;g_{i}=x_{ig}^{⊤}\beta_{g}+\gamma_{g\text{Id}}\;h_{i}=x_{ih}^{⊤}\beta_{h}+\gamma_{h\text{Id}} \end{array}$$

and the vector parameter of interest to be estimated is $\theta =\left(\beta ,\sigma^{2},\Lambda_{\text{Id}},\lambda_{\text{Od}}^{2}\right)$, where


$$\begin{array}{l} \beta =\left(\beta_{a},\beta_{b},\beta_{c},\beta_{d},\beta_{e},\beta_{f},\beta_{g},\beta_{h}\right)\;\text{and}\\ \Lambda_{\text{Id}}=\mathrm{diag}\left(\lambda_{a\text{Id}}^{2},\lambda_{b\text{Id}}^{2},\lambda_{c\text{Id}}^{2},\lambda_{d\text{Id}}^{2},\lambda_{e\text{Id}}^{2},\lambda_{f\text{Id}}^{2},\lambda_{g\text{Id}}^{2},\lambda_{h\text{Id}}^{2},\lambda_{T\text{Id}}^{2}\right) \end{array}$$

with


$$\beta_{◦}=\left(\beta_{0◦},\beta_{1◦},\beta_{2◦},\beta_{3◦}\right)\;\text{for each }◦=\left\{a,b,c,d,e,f,g,h\right\}.$$

Moreover, the observed covariable vector for the *i*-th individual is denoted, respectively, by ***x***_*i*_ = (***x***_*ia*_, ***x***_*ib*_, ***x***_*ic*_, ***x***_*id*_, ***x***_*ie*_, ***x***_*if*_, ***x***_*ig*_, ***x***_*ih*_) with


$$x_{i◦}=\left(1,\text{S}{\text{P}}_{i},{\text{“tP}\prime \prime }_{i},{\text{Start}}_{i}\right)\;\text{for each }◦=\left\{a,b,c,d,e,f,g,h\right\}.$$

Covariate vectors ***x***_*i*°_ record the fixed effects to be tested in each quantity ° = {*a, b, c, d, e, f, g, h*}, in the model for μ, the average response dynamics (i.e. the average reverse detrend TLQ dynamics). In the current context, we restricted the investigation to the treatment's effects (SP and “tP″, being PEI the reference treatment) and also of tinnitus loudness perception at the beginning of the experiment (Start).

#### 4.3.2. Statistical Model Selection and Convergence Criteria

The methods used in this study, to identify convergence from simulated chains, correspond to some graphical and numerical techniques. We include the criteria of Gelman and Rubin (Gelman and Rubin, 1992; Brooks and Gelman, 1998), Geweke (Geweke, 1992), and Heidelberger and Welch (Heidelberger and Welch, 1983). The graphics methods are: histogram and estimated density, which approximates the posterior density; the trace of the resulting chain; limit mean; autocorrelation.

#### 4.3.3. Statistical Model Illustration

The proposed statistical model 3 postulates the expected dynamics of the experiment including stability (or low change) in the response from *T*_1_ to *T*_5_ (where there is no electrical stimulus) followed by a transition period in the *T*_6_; a sigmoidal growth over the remainder online period and finally an exponential decay after the interruption of the electrical stimulation.

**Figure 9:**
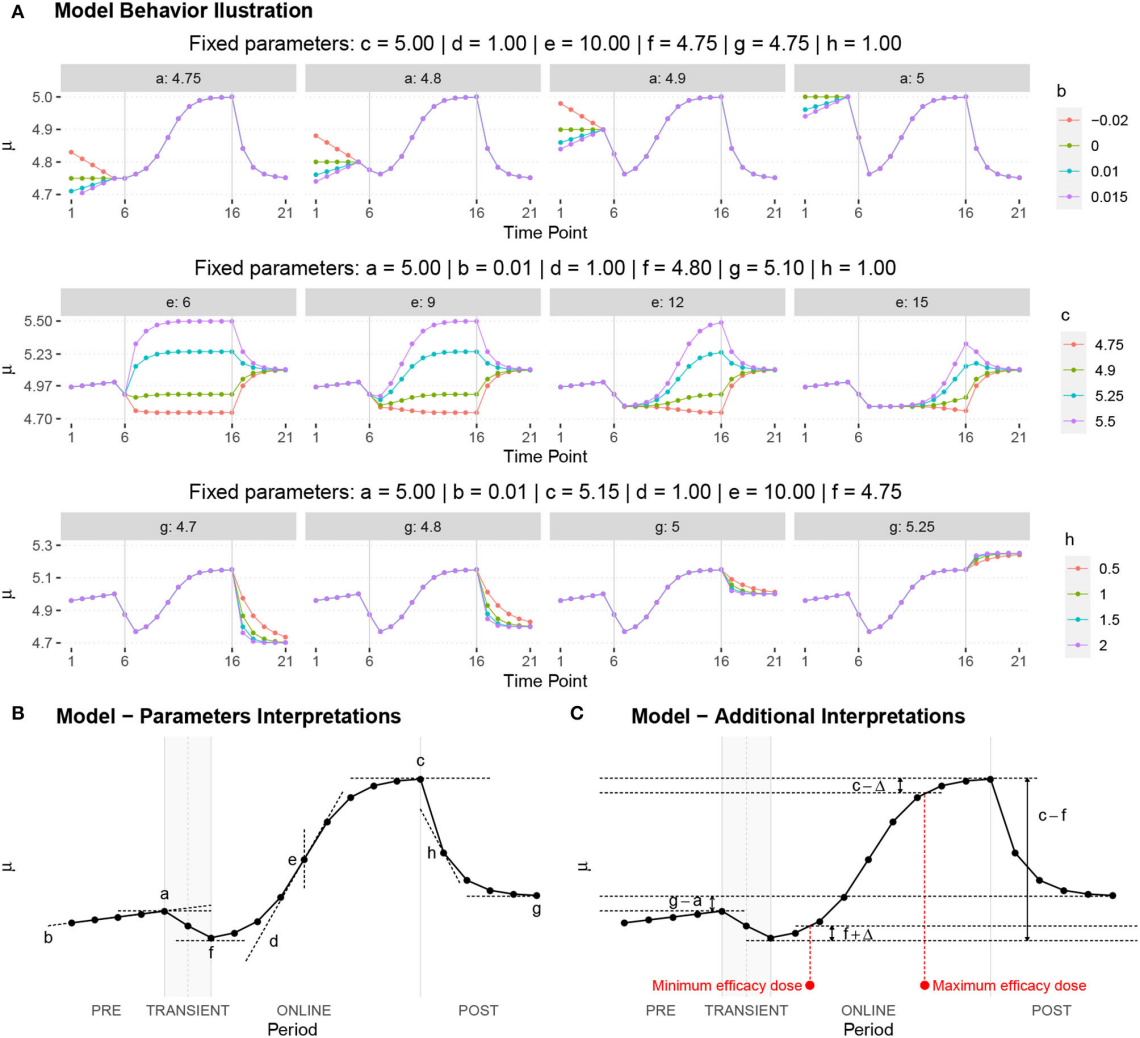
**(A)** Illustration of the proposed model to represent the expected dynamics of the experiment. **(B)** The highly interpretative fundamental model parameters (the parameters {*a, b, c, d, e, f, g, h*}) and some of its functions in **(C)**.

Each fundamental quantity of this model (parameters {*a, b, c, d, e, f, g, h*}), and even some functions of such quantities have a direct interpretation of the average dynamics μ of the studied phenomenon represented by random variable *Y*. Table 1 shows a summary of the main representations also displayed in Figures 9A–C illustrate possible behaviors postulated in this model based on the variation of the parameters.

**Table 1:** Model parameters representations.

	**Parameter**	**Parameter representation**	**Expression**
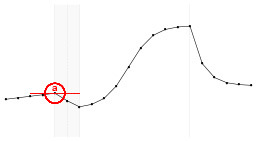	***a***	Represents the μ value at the end of the PRE period	—
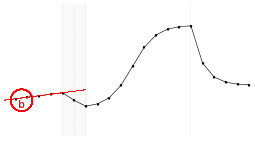	***b***	Represents the rate of change of μ in the PRE period	—
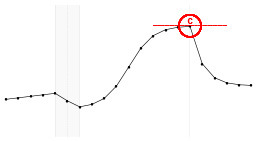	***c***	Represents the maximum value of μ in the ONLINE period	—
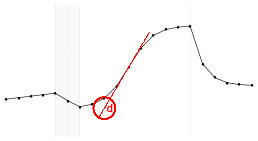	***d***	Represents the intensity of μ growth in the ONLINE period	—
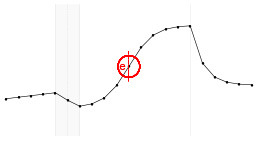	*e*	Represents the inflection point of μ growth in the ONLINE period	—
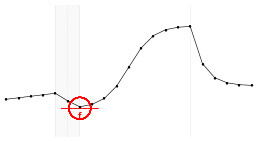	*f*	Represents the minimum value of μ in the ONLINE period	—
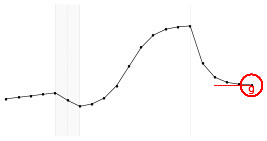	*g*	Represents the minimum value of μ in the POST period	—
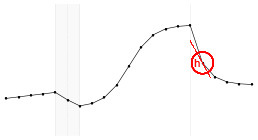	*h*	The *h* parameter represents the intensity of μ decay in the POST period	—
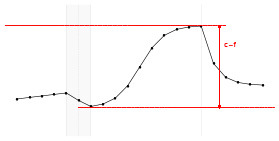	Gain	Represent the potential gain of μ during the online period	*c*−*f*
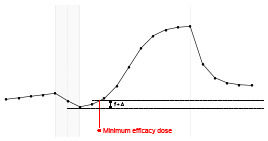	DMin {*j*:μ_·DMin_ = *f*+δ}	Represents the point of the ONLINE period at which treatment reaches the minimum expected effect	$-\frac{1}{d}\log\left(\frac{c-f-\delta }{\delta }\right)+e$
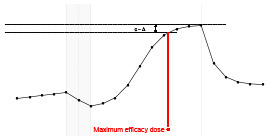	DMax {*j*:μ_·DMax_ = *c*−δ}	Represents the point of the ONLINE period at which treatment reaches its maximum significant effect	$-\frac{1}{d}\log\left(\frac{\delta }{c-f-\delta }\right)+e$
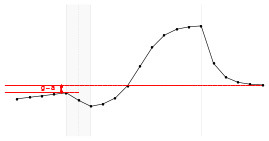	CO	The effect of potential carry-over within the trial period	*g*−*a*

## 5. Results

### 5.1. Adjusted Bayesian Model Interpretation

We assumed that *Y* denotes the observed dynamics (increase or decrease) between the responses during 21-time points (*T*_1_ – *T*_21_) of the session and the sample size is too small for any conclusive statement. Therefore, the result shown here should be seen as indicative, since the uncertainty involved in the estimates is still very large and many criteria for the convergence of the chains have not been met, considering a statistical significance of (0.05/36) ≈ 0.001 resulting from the correction of the significance level by the Bonferroni criterion for the multiple comparisons with a confidence of 95%. Based on the estimates parameters of the statistical model 3, shown in Table 2, the following are the preliminary findings:

- No evidence indicates a difference between treatments on μ final point (*a* parameter), or poor rate of change (*b* parameter) in the PRE period;
- No evidence shows a difference between “tP” and PEI on μ at maximum value during the online period (*c* parameter); however, the “SP” effect was statistically distinct from PEI;
- No evidence showes a difference between treatments on μ response when we observed the intensity of change in dynamics during the ONLINE phase (*d* parameter);
- It was found that “tP” and “SP” treatments were statistically distinct PEI on μ response when we observed a point of change (inflection, e parameter) in the dynamics during the ONLINE period–presenting the change in a lower time point;
- No results demonstrated a difference between treatments on μ response when the minimum value was observed in the ONLINE period (*f* parameter);
- In this study, we could not observe any difference between treatments regarding the lower value reached in the POST period (*g* parameter);
- There was no evidence to show a difference between treatments in the intensity at which μ decays in the POST period (*h* parameter);
- TLQ scale may be associated with the minimum value of μ in the ONLINE period (*f* parameter) and intensity of μ decay in the POST period (*h* parameter).

**Table 2:** Summary measures for the model adjust.

			**Posteriori measures**		**95% Credibility interval**		**Standard error**		**Convergence diagnosis**		
		**Priori distribution**	**Mean**	**SD**	**2.5%**	**97.5%**	**Naive**	**Time-series**	**GR statistics**	**HW *p*-value**	**GW *p*-value**
**a** μ at the end of the PRE period	Intercept	N(5.0,0.1)	4.6802	0.2892	4.1132	5.2554	0.0017	0.0031	1.017	0.0001	0.8726
	PEI	—	—	—	—	—	—	—	—	—	—
	“SP”	N(0.0,0.25)	-0.0395	0.1867	–0.4118	0.3263	0.0011	0.0011	1.012	0.0351	0.7918
	“tP”	N(0.0,0.25)	0.1082	0.1959	-0.2927	0.4832	0.0011	0.0017	1.114	0.0000	0.0255
	Start	N(0.0,0.001)	0.0663	0.0336	–0.0008	0.1316	0.0002	0.0004	1.019	0.0003	0.8315
**b** Rate of change of μ in the PRE period	Intercept	N(0.0,0.1)	–0.0904	0.1196	–0.3247	0.1457	0.0007	0.0011	1.013	0.0001	0.9594
	PEI	—	—	—	—	—	—	—	—	—	—
	“SP”	N(0.0,0.25)	–0.0304	0.0776	–0.1831	0.1227	0.0004	0.0005	1.010	0.0964	0.8813
	“tP”	N(0.0,0.25)	0.0384	0.0803	–0.1247	0.1929	0.0005	0.0005	1.074	0.0001	0.0286
	Start	N(0.0,0.001)	0.0178	0.0139	–0.0097	0.0450	0.0001	0.0001	1.015	0.0000	0.8052
**c** Maximum value of μ in the ONLINE period	Intercept	N(4.0,1.0)	3.2523	1.5190	0.5685	6.1414	0.0088	0.0576	2.308	0.9985	0.3266
	PEI	—	—	—	—	—	—	—	—	—	—
	“SP”	N(0.0,0.25)	**0.5594**	0.3193	**0.0137**	**1.2259**	0.0018	0.0101	1.201	0.0000	0.9015
	“tP”	N(0.0,0.25)	0.6892	0.4051	–0.0710	1.4673	0.0023	0.0117	1.625	0.0000	0.4951
	Start	N(0.0,0.001)	0.4256	0.3554	–0.1951	0.8308	0.0020	0.0325	5.419	0.0418	0.8857
	$\lambda_{2}^{c\text{ Id}}$	GA(0.001,0.001)	10.6178	14.6583	0.6263	51.6616	0.0846	0.6040	1.273	0.0000	0.8797
**d** Intensity of μ growth in the ONLINE period	Intercept	N(2.0,0.25)	0.8741	2.1280	-2.5344	5.2837	0.0123	0.1831	3.325	0.0001	0.9261
	PEI	—	—	—	—	—	—	—	—	—	—
	“SP”	N(0.0,0.25)	0.0852	0.8082	–1.1995	2.4646	0.0047	0.0804	1.327	0.0001	0.6211
	“tP”	N(0.0,0.25)	–0.4653	1.2676	–2.6402	2.8200	0.0073	0.1254	2.086	0.0012	0.5072
	Start	N(0.0,0.001)	0.0387	0.3646	–0.8363	0.6301	0.0021	0.0373	2.924	0.0042	0.9309
**e** Inflection point of μ growth in the ONLINE period	Intercept	N(11.0,1.0)	13.0446	0.7350	11.7258	14.6473	0.0042	0.0206	1.033	0.0001	0.5595
	PEI	—	—	—	—	—	—	—	—	—	—
	“SP”	N(0.0,0.25)	**–4.6069**	0.9658	**–6.1306**	**–1.7550**	0.0056	0.1005	1.416	0.0116	0.7126
	“tP”	N(0.0,0.25)	**–4.7487**	1.4181	**–6.8166**	**–0.9649**	0.0082	0.1309	1.257	0.0009	0.5210
	Start	N(0.0,0.001)	0.0811	0.2990	–0.7794	0.4869	0.0017	0.0317	1.329	0.0000	0.6371
	$\lambda_{2}^{e\text{ Id}}$	GA(0.001,0.001)	32.5357	50.0955	—	177.1386	0.2892	0.6062	1.025	0.3637	0.7693
**f** Minimum value of μ in the ONLINE period	Intercept	N(4.5,2.0)	3.6517	0.7266	2.3064	5.1378	0.0042	0.0116	1.066	0.0001	0.3857
	PEI	—	—	—	—	—	—	—	—	—	—
	“SP”	N(0.0,0.25)	0.1124	0.2505	–0.3005	0.6905	0.0014	0.0055	1.412	0.0000	0.8875
	“tP”	N(0.0,0.25)	0.0076	0.4141	–0.5781	0.8738	0.0024	0.0236	3.514	0.0000	0.4468
	Start	N(0.0,0.001)	**0.4573**	0.2240	**0.1573**	**0.9062**	0.0013	0.0119	2.930	0.0008	0.6930
	$\lambda_{2}^{f\text{ Id}}$	GA(0.001,0.001)	17.8428	23.1012	1.0976	79.1864	0.1334	0.4549	1.686	0.0000	0.3144
**g** Minimum value of μ in the POST period	Intercept	N(5.0,0.1)	5.3116	1.0513	3.3257	6.4368	0.0061	0.0310	5.884	0.0001	0.8223
	PEI	—	—	—	—	—	—	—	—	—	—
	“SP”	N(0.0,0.25)	0.0317	0.2043	-0.2915	0.5173	0.0012	0.0075	2.004	0.0687	0.7787
	“tP”	N(0.0,0.25)	–0.2654	0.2477	-0.6331	0.2305	0.0014	0.0018	3.083	0.6585	0.9370
	Start	N(0.0,0.001)	-0.0273	0.1042	–0.1392	0.1810	0.0006	0.0041	4.797	0.0000	0.7354
**h** Intensity of μ decay in the POST period	Intercept	N(0.0,0.1)	–3.0260	1.9187	–5.7671	1.9543	0.0111	0.1134	1.621	0.8183	0.9474
	PEI	—	—	—	—	—	—	—	—	—	—
	“SP”	N(0.0,0.25)	2.6748	2.8473	–3.4865	6.1107	0.0164	0.0606	4.210	0.0059	0.7906
	“tP”	N(0.0,0.25)	1.6385	1.9728	–3.2636	4.8426	0.0114	0.0136	1.894	0.0000	0.9354
	Start	N(0.0,0.001)	**2.9808**	8.0595	**0.4253**	**29.1606**	0.0465	0.6893	1.438	0.7807	0.5146
	σ^2^	GA(0.001,0.001)	0.1975	0.0368	0.1497	0.2757	0.0002	0.0008	3.621	0.0000	0.8235

*In all simulated contexts, R software (R Core Team, 2020) was used with RStudio interface support of some packages; The models and the simulated samples of the posterior distribution were obtained with the packages jags (Plummer, 2018) and coda (Plummer et al., 2006); The tests used for chain diagnosis are built-in coda packages and consider the multiple comparisons of convergence of the chains using the Bonferroni correction of the significance level; A total of 50,000 iterations were considered as adaption period for simulation methods. 100,000 iterations for burn-in and 50,000 final samples performed with 50-in-50 jumps to simulate an independent sample; All simulations were performed on an Intel(R) Core i7-7700HQ CPU 2.80 GHz with 8 GB of RAM. The bold items indicate the proof of significance of the each parameters factor*.

Note that the estimate for the variance of the intra-individual deviations ε_*ij*_ = *y*_*ij*_ − μ_*ij*_ is given by ${\hat{\sigma }}^{2}=0.1975$ (with a standard deviation near to 0.37), indicating low intra-individual variability. On the other hand, an indication of the curve variation between individuals can be perceived based on the estimates for the mean and the standard deviation of $\lambda_{c\text{Id}}^{2}\left(10.62;\;14.66\right)$, $\lambda_{e\text{Id}}^{2}\left(32.54;\;50.10\right)$ and $\lambda_{f\text{Id}}^{2}\left(17.84;\;23.10\right)$. We noticed that among individuals, there is significant variation in the highest and lowest average value reached in the ONLINE period (parameters *c* and *f*, respectively) and also the inflection point of the growth curve (parameter *e*).”

**Figure 10:**
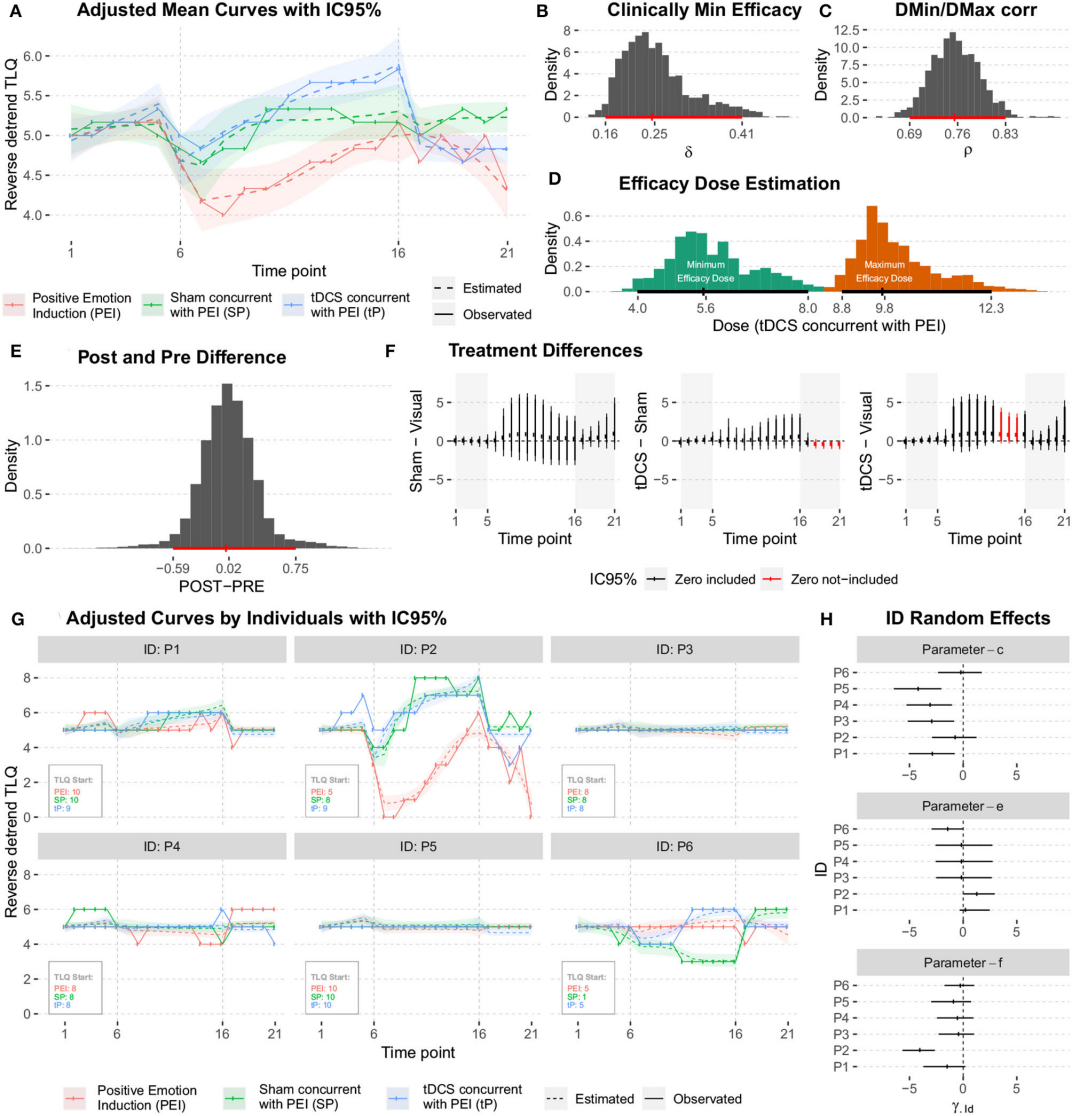
**(A)** Graphical summary for the mean curve, showing the adjusted and observed behaviors. **(B)** The posteriori distribution of the Minimum Clinical Efficacy (δ). **(C)** The posteriori distribution of the correlation between μ_DMin_ and μ_DMax_. **(D)** The posteriori distributions of the minimum and maximum effective doses. **(E)** The posteriori distribution for the difference between the average responses during the POST (*T*_21_) and PRE (*T*_1_) periods. **(F)** The posterior distributions of the difference between treatments (conditioned to the time point). **(G)** The mean curve dynamics considering the individual random effects predictions. **(H)** The posteriori distributions for the predictions were obtained for ID random effects.

A general graphical summary of the mutual interaction of all parameters in the form of their mean curves is depicted in Figure 10A. It displays the adjusted and observed behaviors also. The treatment curves are constructed based on the means estimates from posterior distributions for each studied effect, including the averages predicted for random effects (Figure 10H).

The average dynamics can be described according to the predictions of individual random effects ${\hat{\gamma }}_{i\text{Id}}$ and ${\hat{\gamma }}_{i\text{Or}}$, This gives details of the estimates for each studied individual. The results are shown in Figure 10G.

The predictions obtained for ID random effects (Figure 10H) indicate high variability among subjects, and show intrinsic characteristics to the studied individuals that must be taken into account in the modeling. In particular, the higher variability was observed in individuals' random effects for *c* and *f* parameters.

The posterior distributions of the quantities of interest must be observed to address further specific questions. No significant difference was observed in the average responses during post- and pre- periods. No significant difference between the two responses was observed as they were distributed around zero with low variability (Figure 10E).

Investigation of the hypotheses about the effects of the treatments on the average response during 21-time points of the session; A summary of the posterior distributions of the difference between treatments (conditioned to the time point) is shown in Figure 10F. Due to the high variability (probably because of the low sample size), no significant difference (Minimum Clinical Efficacy, δ) is observed between the treatments.

A summary of the posterior distributions of the difference between treatments (conditioned to the time point) is shown in Figure 10F. Due to high variability (probably because of low sample size), no significant difference [Minimum Clinical Efficacy (δ)] was observed between different treatments. We got posteriori distributions of δ (see Figure 10B), the correlation between μ_DMin_ and μ_DMax_ (Figure 10C), and minimum and maximum effective doses (Figure 10D) for “tP” treatment, calculated by the equations for **Dmin** and **Dmax** (as show in Figure 10D and Table 1). Several results showed that were pieces of evidence the minimum effect of the treatment was reached around time point ***T***_**6**_, and the maximum effect was obtained near time point ***T***_**10**_.

The adjusted model provided positive outcomes and enabled the extraction of vital information to determine the proper sample size for the testing of hypotheses with the desired credibility. Both the model and the appropriate equation for sample size calculation provided some information from “sample sizes” distributions. The following section reports the results.

### 5.2. Sample Sizes Determination for Seamless Adaptive Study Design

**Figure 11:**
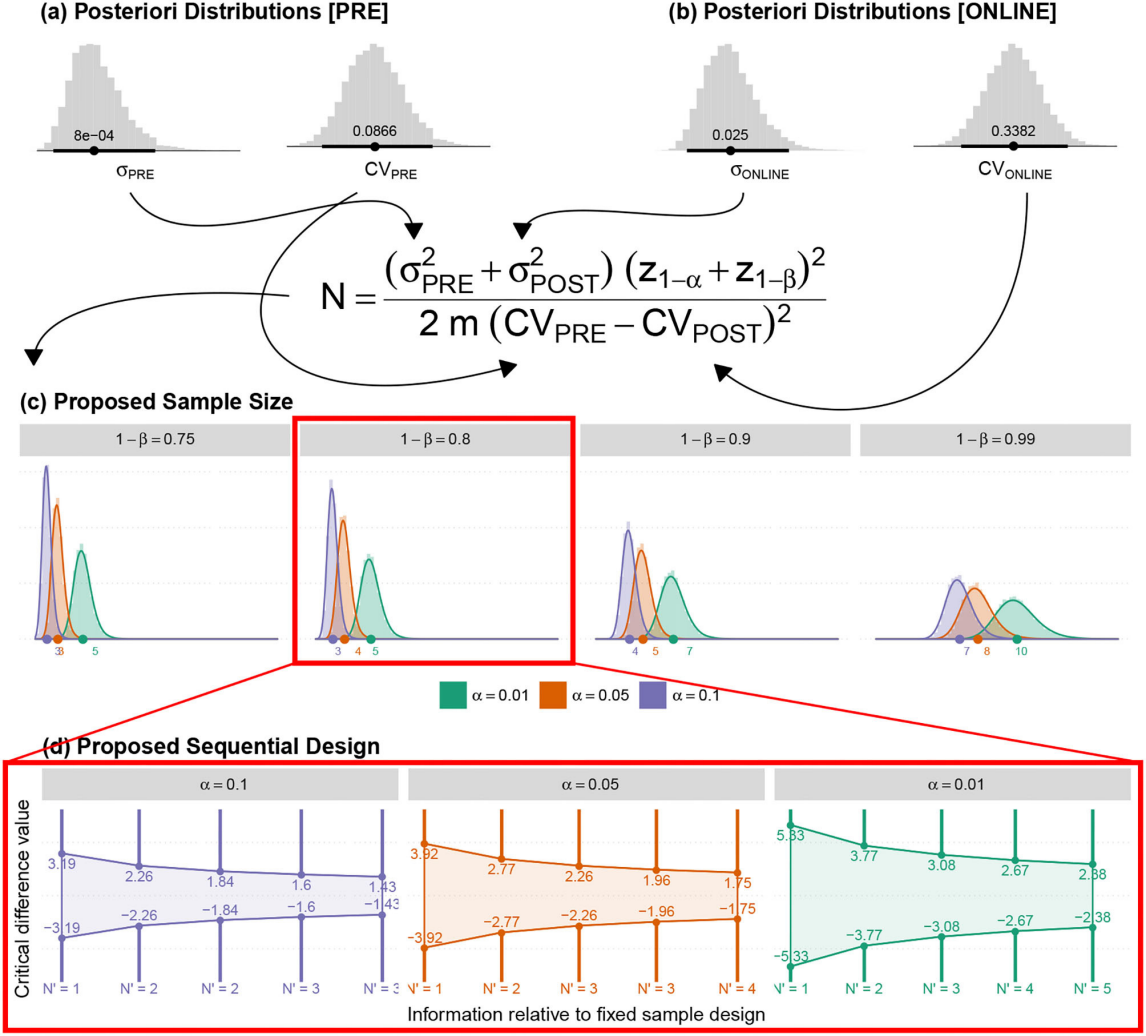
**(a,b)** The posteriori distributions for the variances and Coefficient of Variance (CV) for the average responses (associated with the Pre and Online blocks) were obtained by adjusting the proposed model. **(c)** The posteriori distribution of the new sample sizes (based on fixed α, β and *m*). **(d)** The suggested sequential sampling plan.

**Figure 12:**
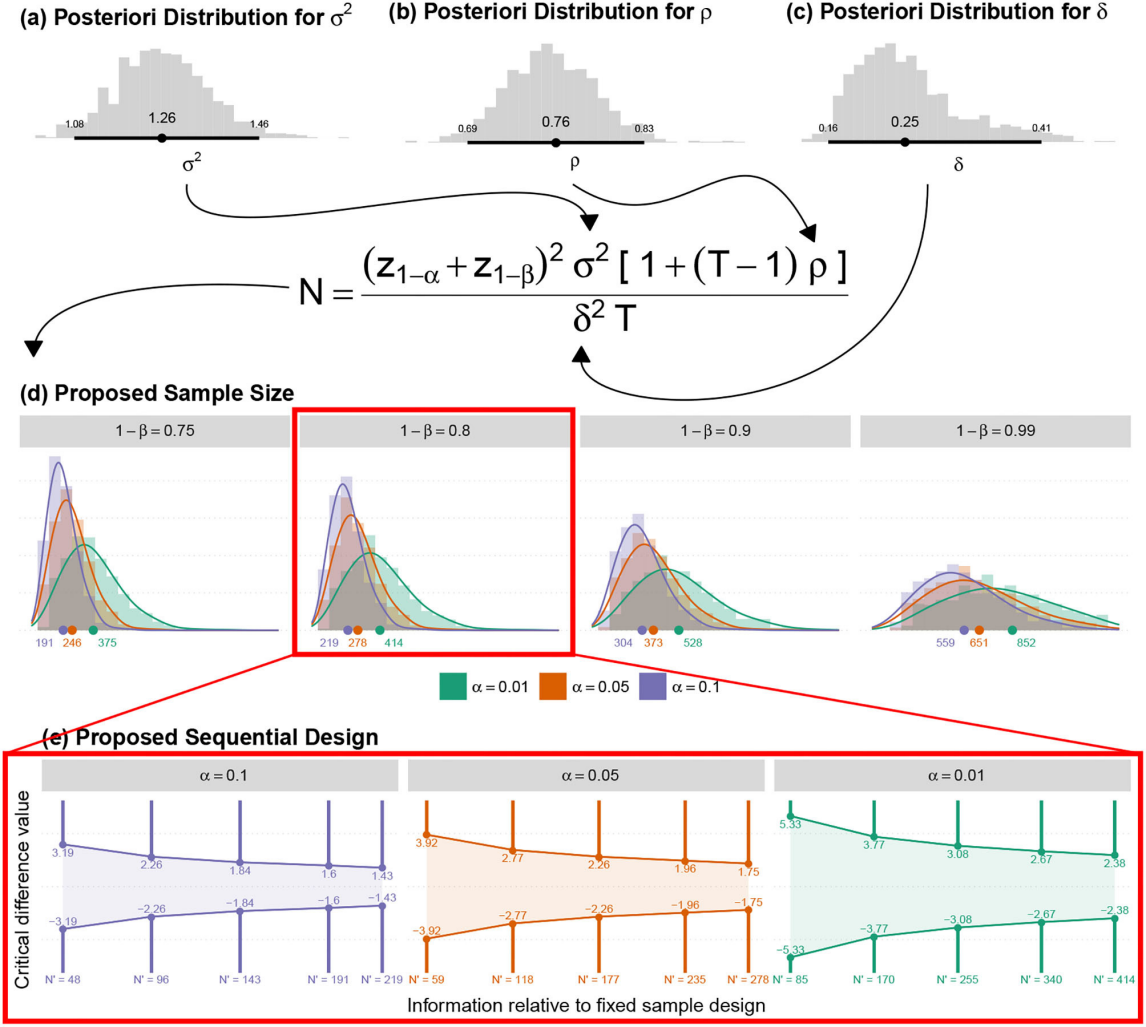
**(a)** The posteriori distribution for the marginal variance of the adjusted model; **(b)** The posteriori distribution of the correlations, obtained by the average correlations between all-time points. **(c)** The posteriori distribution of the Minimum Clinical Efficacy was obtained with the adjusted model. **(d)** The posteriori distribution of the new sample sizes (for all other fixed amounts). **(e)** The suggested sequential sampling plan.

**Figure 13:**
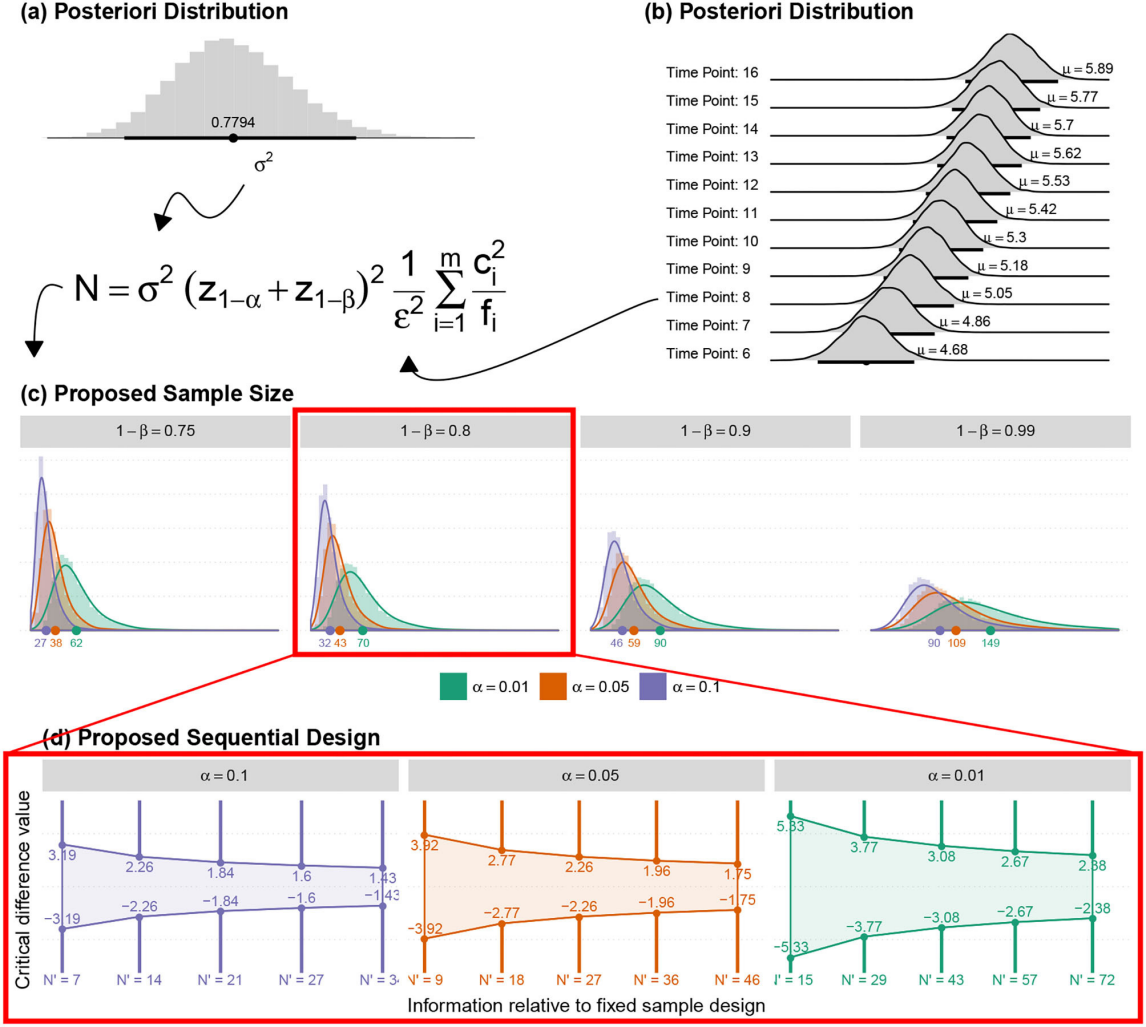
**(a)** The posteriori distribution for the marginal variance of the adjusted model (just on Online block); **(b)** The posteriori distribution of the mean response for each online time point; **(c)** The posteriori distribution of the new sample sizes (for all other fixed amounts); and **(d)** The suggested sequential sampling plan.

Proposed actions after adjusting the model incorporated estimated numbers that suggested the sample sizes (as illustrated in Figure 6) after considering the actions *N*_3_, *N*_4_, and *N*_5_ in determining sample size. We employed three seemingly adequate expressions for our situation (Figures 11–**13**) for the sample sizes derived from the studies (Twisk, 2013; Chow et al., 2017). We established these expressions as functions of random variables derived from the proposed statistical model 3 with their posteriori distributions estimated using the Bayesian approach. Therefore, we propose sample sizes based on random variables (*N*_3_, *N*_4_, and *N*_5_) and corresponding estimated distributions.

#### 5.2.1. (*N*_3_) Identification of the Difference Between Pre-and-Online-Blocks in Active-Control–PEI

Since there were no prior PEI studies undertaken on the tinnitus population in the literature, we calculated the minimum sample size to ensure the efficacy of the applied active control. The estimation was based on the Coefficient of Variances (CVs) of Pre block against the online block in PEI treatment. Besides, sample CV for the five PRE time points per individual (CV_PRE_ = 0.0598) and for the first five ONLINE time points per individual (CV_ONLINE_ = 0.367) were also employed. Table 3 depicts the summary of the results using the illustrated equation in Figure 11.

**Table 3:** Sample size based on usual approach to identify difference between CV of PRE and ONLINE blocks of PEI treatment.

	**Significance**		
**Power (%)**	**1%**	**5%**	**10%**
75	4	3	2
80	4	3	3
90	6	4	3
99	9	7	6

According to the proposed methodology and the adjusted data-model, the posteriori distributions of the quantities necessary for adequate sample size were determined that led to the estimation of the posterior distribution of the suggested sample size. A sequential sampling plan was then proposed. Both sample sizes (*N*) and Minimum Clinical Efficacy (δ) can be adapted in each intermediate stage according to the preliminary results.

#### 5.2.2. (*N*_4_) Identification of Differences Between Treatment Effectiveness

A linear model for repeated measures was adjusted over reverse-detrend TLQ observations during the online period considering a random intercept per individual. As a result, the point estimate of intra-individual variability $\sigma_{0}^{2}\;\approx \;0.08731$, residual variability σ^2^ ≈ 1.09283, and intra-class correlation coefficient (indicative of the correlation between repeated measures) were given by $\rho =\sigma_{0}^{2}/\left(\sigma_{0}^{2}+\sigma^{2}\right)\approx 0.07398$ though any simple model could calculate variability but the proposed model is remarkable as it facilitates immediate estimation of ρ impractical). The proposed sample sizes are illustrated in Table 4.

**Table 4:** Required sample sizes to identify significant difference between treatment effects.

	**Significance**		
**Power (%)**	**1%**	**5%**	**10%**
75	17	11	9
80	19	13	10
90	24	17	14
99	39	30	25

Figure 12 displays the posteriori distributions for sample sizes (for fixed significance, α, and power, 1 − β) calculated under the following considerations:

- The measure (ρ) indicates the correlation between the simulated average responses at DMin and DMax time-points or Corr(μ_DMin_, μ_DMax_) as shown in Figure 12;
- The overall estimated posteriori distributions were employed to estimate the marginal variance of the response variable regardless of each particular time-point. Thereafter, the calculation of the sample size at each time-point was enumerated (The expression is shown in Figure 12).

#### 5.2.3. (*N*_5_) Estimation of the Sample Size for Investigating Minimum and Maximum Effective Dose

The sample means for the 11 ONLINE time-points (${\hat{\mu }}_{6}=5$, ${\hat{\mu }}_{7}=4.83$, ${\hat{\mu }}_{8}=5$, ${\hat{\mu }}_{9}=5.17$, ${\hat{\mu }}_{10}=5.17$, ${\hat{\mu }}_{11}=5.5$, ${\hat{\mu }}_{12}=5.67$, ${\hat{\mu }}_{13}=5.67$, ${\hat{\mu }}_{14}=5.67$, ${\hat{\mu }}_{15}=5.67$, ${\hat{\mu }}_{16}=5.83$), the sample variance in this period (${\hat{\sigma }}^{2}=0.547$) and the contrasts (*c*_6_ = −4, *c*_7_ = −4, *c*_8_ = 0.5, *c*_9_ = 0.5, *c*_10_ = 1, *c*_11_ = 1, *c*_12_ = 1, *c*_13_ = 1, *c*_14_ = 1, *c*_15_ = 1, *c*_16_ = 1) were used for a conventional calculation of the sample sizes (Figure 13a) shown in Table 5.

**Table 5:** Required sample sizes to identify significant difference between treatment effects.

	**Significance**		
**Power (%)**	**1%**	**5%**	**10%**
75	49	29	21
80	54	34	25
90	71	47	36
99	117	85	71

On the other hand, the ASB method employed the posteriori distributions for the mean response and variance to determine the sample sizes' posterior distributions required for the identification of minimum and maximum effective doses. The contrasts were also obtained, according to the minimum and maximum effective dose estimated with the proposed model.

### 5.3. Application of the Adaptive-Seamless Bayesian Methodology

Adaptive-Seamless Bayesian (ASB) methodology was employed for the current study design. Possible successive adaptations for sufficient sample sizes were considered for the achievement of the desired power level validating hypotheses associated with statistically significant efficacy of a given treatment and the difference between treatments. In other words, ASB benefits Bayesian and adaptive advantages in a single study design.

#### 5.3.1. Adaptive Methodology

An adaptive clinical study within the design stage facilitates prospective correctional modification based on aggregating interim data from subjects during the study. Correctional modifications include, but are not limited to the suggestion of sequential trials and iterative re-estimate of sample sizes, which remain the focus of this study. This may be advantageous for both clinicians and financers as performing sequential trials is common in the current standard approaches. It gives the scope of the interpretation (for efficacy or futility) based on interim analysis results. The adaptive process is based on partially observed results regardless of the proposed sample size. In the adaptive context, interim analyses result in verifying efficiency or futility, thus highlighting the need for further testing sequence. A necessary readjustment of the sample size may be adapted to the subsequent interim analysis, which is prohibited in conventional approaches without prior approval by the Ethics committee. Furthermore, a seamless adaptive trial design combines two independent trials in a single study. The study objectives can be clarified from individual studies. Before adaptation, a seamless adaptive design incorporates two stages: initially before adaptation (exploratory- stage) and after adaptation (confirmatory-stage). The exploratory-stage intends to explore the tests of uncertainty factors in treatments to apply adaptation. It also permits investigators to halt the trial beforehand due to single or multiple concerns related to safety, futility, and accrued data efficacy. The confirmatory-stage may validate preliminary findings from the exploratory-stage. The most significant advantage of the two-stage adaptive seamless is the merging of accumulated data from both stages toward more accurate and reliable inferences.

#### 5.3.2. Bayesian Methodology

Bayesian methods show features of adaptability through self-idealization, by assuming a priori knowledge that can be updated with new information and application to several contexts. Furthermore, it enables even subjectively the uncertainty in any parameter, which may be ignored by traditional methods. In classical methods, the use of fixed expression quantities to determine sample size leads to a point determination of sample size, ignoring possible variability in the estimation processes. For example, the determination of the sample size by the equation as shown in Figure 12. In such a context, values must be provided for σ^2^ and ρ, which are uncertain quantities when σ^2^ and ρ summarized by their point estimates from a pilot study. In this scenario, the Bayesian approach utilizes the information of the corresponding probability distributions related to σ^2^ and ρ. Such distributions can be expressed in terms of either the researcher's subjective knowledge based on experience (a priori knowledge) or the results from a preliminary study (a posteriori distribution of a pilot study becomes a priori knowledge at any stage after it) that preserves all possible variances in low sampling. Conventional methods generally ignore all variances of low sampling.

#### 5.3.3. The Joint Use of the Two Approaches

The use of adaptive methods in a Bayesian context shows potential for analysis with many advantages over conventional methods. Among them, the natural control over uncertainties of desired quantities includes mean values and their possible differences, inter/intra-subject variability, power (like 1 − β calculated by the equation shown in Figure 12), size of the effect (isolating δ or δ/σ in the equation shown in Figure 12), and the sample size itself. All desired quantities can be described by a probability distribution (a posterior distribution) that includes information adapted from a priori distribution, both subjective (in terms of expert's perception) or objective (based on preliminary studies). In the current study, the sample sizes were calculated according to probability distributions that consider the variability involved in the estimation processes of σ^2^, CV*s*, ρm μs from the pilot study. The posteriori distributions for σ^2^ and ρ were employed to determine *N*_4_. Specific aspects of the experimental design (the *T* and δ) together with the estimated distribution provide plausible sample sizes according to criteria of interest (significance α and power 1 − β).

#### 5.3.4. Computation of the Progressive Uncertainty Reduction

The new information enables the updation of the results from earlier phases at each interim. Hence, the establishment of a procedure that quantifies the credibility of the previous phase depends on the length of credibility interval of the relatively uncertain quantity. The proposed method is applied to all desired parameters in the study such as minimal clinical efficacy, the maximum and minimal efficacy dose, and all necessary sample sizes:

1. Considering the posterior distributions of stages *i* − 1 and *i*, we evaluated whether the expected values of the two distributions would be considered equivalent using the Bayes Factor. It assesses the degree of relative evidence of the results from stage *i* − 1 compared to the results from stage *i*, that are embedded in the new information;
2. If the values expected in both stages are considered equivalent, we can determine the respective lengths of the credibility intervals (at a given arbitrary level);
3. The R ratio between the lengths obtained in stages *i* and *i* − 1 are determined. Quantity UR = (1 − R) × 100% is interpreted as the percentage of **“uncertainty reduction”** in stage *i* − 1, since the information obtained in stage *i* is considered;
4. In normal situations, the increase in the sample size in the intermediate stages may consistently reduce the UR measure until the stability UR has neared zero.

**Figure 14:**
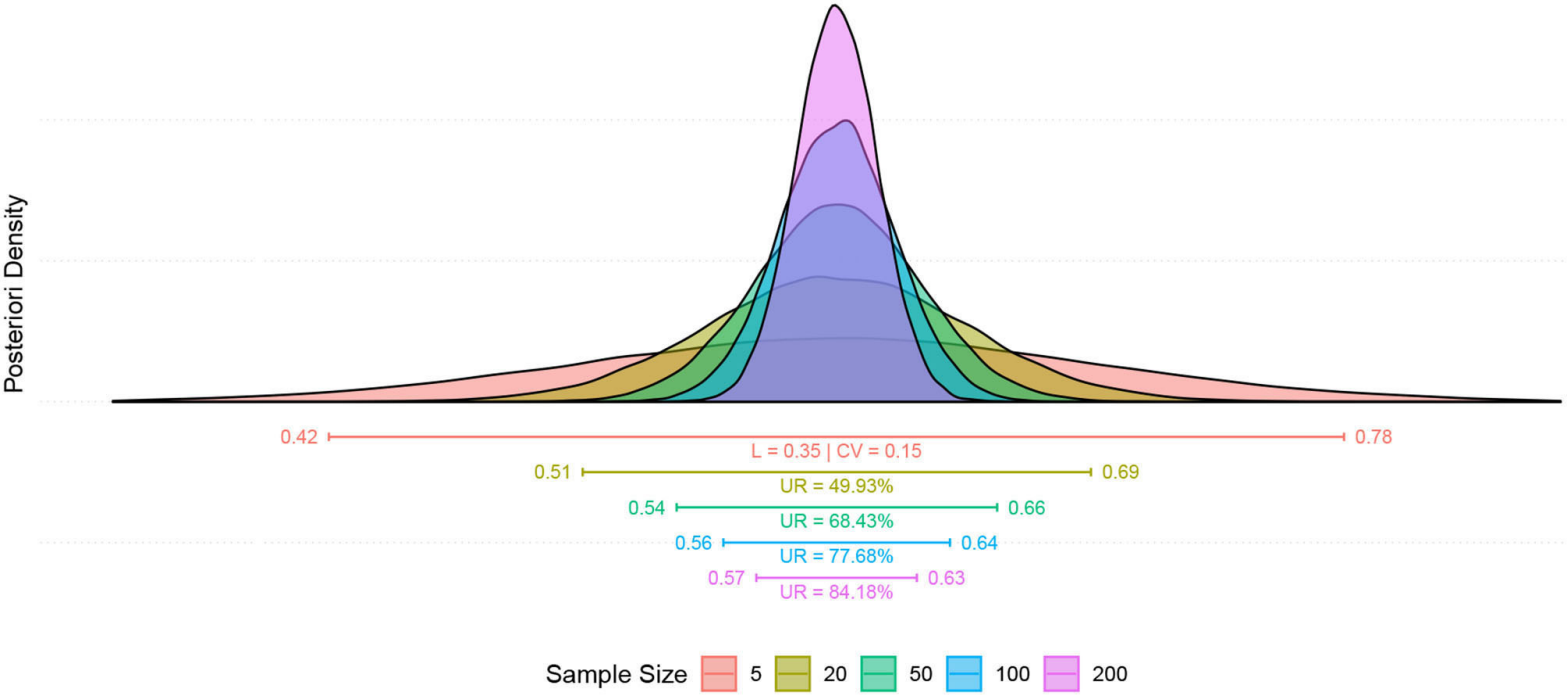
Illustration (in a simulated context) of the progressive uncertainty reduction due to increased sample size. The first segment (in red) highlights the length of the credibility interval (L) and the coefficient of variation of the a posteriori distribution (CV), in the following segments the magnitude of UR for each stage stands out (*n* = 20, 50, 100, 200) when compared to the uncertainty we had in the first study (*n* = 5).

Another possibility of quantifying the behavior of uncertainty reduction can be based on the percentage reduction of the coefficient of variation associated with the posterior distributions. The length of the interval (in step 2) can be replaced with the coefficient of variation. Figure 14 illustrates the expected behavior of the posterior distribution based on the progressive increase in the sample size. The UR measure stabilizes at the point at which the amount depends on the phenomenon studied.

## 6. Discussion

In a nutshell, FDA recommends that statistical inferences on efficacy and safety should be performed based on the responses of the primary study endpoints collected from a sufficient number of subjects while employing suitable statistical methods originated from the study design and objectives to accomplish a well-controlled study. This study proposes a novel Adaptive-Seamless Bayesian (ASB) methodology for HD-tDCS Dose-Response Study Design that facilitates the long journey of neuromodulation from a research protocol to the clinical recommendation and treatment individualization. The method was prototyped for six tinnitus patients as a pilot to elucidate the tinnitus negative valence role. Here below, we discuss the advantages, challenges, and future trends of the protocol and ASB methodology.

### 6.1. Protocol Advantages and Challenges

Neuromodulation research is complicated on account of the involvement of cognitive processes and brain networks. Therefore, the development of a testable framework such as a Neurofunctional Tinnitus Model (NfTM) is essential for simplifying the brain complexity and providing a roadmap to find a proper research approach, an analytical method, and procedures for the research question. In the presented prototype, the use of the Neurofunctional Tinnitus Model improved our knowledge and enabled the right prediction of cognitive impairments and their rehabilitation. In light of the Neurofunctional model predictions, hypotheses are formulated to measure clinical endpoints.

- Despite its accessibility, affordability, and flexibility, the HD-tDCS neuromodulation technique has some limitations in its methodologies and potential applications. Primarily, anatomical and functional targeting on the brain is required to boost the neuromodulation effect. For anatomical targeting, we developed an atlas-based head-model for the pilot study. Head-model can be individualized in more advanced parts of the journey. Positive emotion induction (PEI) technique was used for functional targeting, and to provoke positive emotional processing of Left dlPFC to reduce tinnitus negative valence.
- Extra modules of the designed study support investigations on neuro markers, biomarkers, surrogate endpoints, and objective measurement factors throughout dense-array EEG, emotional Stroop task, functional and structural MRI as well as a battery of questionnaires.
- Dose parameters including waveform, polarity, intensity, duration, electrode sizes, and montages, regardless of the flexibility in the dose design, can induce complexities as well as unexplored factors.

### 6.2. Advantages and Challenges of Method

Neuromodulation studies with HD-tDCS clinical trials have similar limitations as in a well-controlled studies. Therefore, ASB plays a pivotal role in addressing the problems by employing the following:

1. Bayesian approach to control type I error and improve power;
2. The adaptive methodology to prevent misusing patient resources for investigating undesired doses, and
3. To finalize the trial when it is sufficiently clear that the neuromodulation technique is inefficient;
4. Seamless to lead to the confirmatory phase when the evidence in the exploratory phase infers the requirement;
5. Adapting the ongoing study to the actual variability in the accumulating data for shortening or postponing the study termination;
6. Exploring a longitudinal surrogate endpoint in predicting final results for patients with incomplete data in the carried out pilot based on neurofunctional tinnitus model to predict the loudness misperception.

### 6.3. Future Trends in Protocol and Clinical Study Methodologies

- Updating the head-model is essential to adapt the methodology for other neuromodulation techniques (transcranial Electrical Stimulation, transcranial Magnetic Stimulation, and low intensity focused ultrasound Rezayat and Toostani, 2016).
- ASB can support individualized treatment planning according to the nature of the Bayesian approach.
- To improve functional targeting in needed large scale study concurrent with neuromodulation for improving the efficacy.
- Functional targeting can adapt to each patient individually.

Further validation, particularly under the systematic procedure of simultaneous tES-fMRI (Ekhtiari et al., 2022) of the proposed methodology, is suggested in future studies.

## 7. Conclusion

This study proposed a novel model to target tinnitus. In the proposed methodology, during the induction of the functional targeting under HD-tDCS montage and dose-finding process, simultaneously neuromodulation efficacy of the intervention was investigated. The continuous updation of prior knowledge adapts anticipated dose-response and simulated curve with the longitudinal model to define the minimum and maximum effective doses resulting in the superiority effect of the neuromodulation approach.

The results may be promising with a highly effective dose due to transforming the study for the development of a standard randomized confirmatory trial in which active HD-tDCS protocol is compared with a Sham trial (placebo-like).

The establishment of the HD-tDCS intervention protocol with the corresponding functional targeting as an effective methodology can achieve powerful evidence for a regulatory agency if the confirmatory trial validates the effectiveness. The principal disadvantages of the suggested methodology are limited to the requirement of continuous computational analysis and a sophisticated logistic management system.

Overall, the current paper will be very useful and enlightening for scholars and professionals to devise better diagnoses and interventions. It would also help improve existing methodologies thereby reducing the severity and associated disorders of tinnitus. We also highlight the need for large controlled studies for better clinical efficacy and improved outcomes.

## 8. Data Availability Statement

The materials used during this study are available with the corresponding author on reasonable request and filling out NEL-Consent redirecting to Zenodo for access:

- Datasets; 10.528/zenodo.4554504.
- 3D-Simulation of clinical space and environment: 10.528/zenodo.4554510.
- R-toolbox for dose-response sample size calculation: 10.528/zenodo.4554516.
- Superlab protocols: 10.528/zenodo.4554508.

## Ethics Statement

The studies involving human participants were reviewed and approved by Hospital das Clínicas de Ribeirão Preto, University of São Paulo, Brazil (HCRP No. 115155716616.1.1001.5440). The patients/participants provided their written informed consent to participate in this study.

## Author Contributions

IG: leading author responsible for manuscript development, concept and study design, analogy and numerical methodology design, superlab design, clinical data acquisition, and data pre-processing. OG: collaborated in manuscript development, statistical modeling, and analysis. ZV: collaborated in manuscript development, concept and study design, conceptual modeling participation, and data acquisition in the clinic. ADe: collaborated in manuscript development, supervising data mining, and modeling. BM: collaborated in manuscript development, MRI-co-registered head model implementation, and EEG analysis. ADa: collaborated in manuscript development and tDCS HEAD model design. CT: collaborated in manuscript development and tDCS head model implementation in figures and movies. MH: monitoring clinical data acquisition and collaborated in manuscript development. AS: collaborated in manuscript development, MRI and fMRI protocol design, and data acquisition. FL: collaborated in manuscript development, supervised statistical analysis, and data models. JL: supervised clinical data acquisition and conceptual development and collaborated in manuscript development. All authors read and approved the final manuscript and corresponding author.

## Funding

This research is part of a Multidisciplinary Cognitive Rehabilitation (MCR) Platform and was supported (grant no. 2013/07375-0) by Innovation and Diffusion of Mathematical Sciences Center Applied to Industry (CEPID-CeMEAI) of São Paulo Research Foundation (FAPESP). It also supported with Centro de Engenharia Aplicada a Saúde (CEAS) of the University of São Paulo, and the Coordenação de Aperfeiçoamento de Pessoal de Nível Superior (CAPES) for student's scholarship.

## Conflict of Interest

ADa and CT were employed by the company Soterix Medical Inc. The remaining authors declare that the research was conducted in the absence of any commercial or financial relationships that could be construed as a potential conflict of interest.

## Publisher's Note

All claims expressed in this article are solely those of the authors and do not necessarily represent those of their affiliated organizations, or those of the publisher, the editors and the reviewers. Any product that may be evaluated in this article, or claim that may be made by its manufacturer, is not guaranteed or endorsed by the publisher.

## Abbreviations

“SP”: “Sham concurrent with PEI”
“tP”: “tDCS concurrent with PEI”
δ: Minimum Clinical Efficacy
Ar: arousal-ratio
ASB: Adaptive-Seamless Bayesian
BA: Brodmann areas
CAAP: Conscious Attended-Awareness Perception
CV: Coefficient of Variance
dlPFC: Dorsolateral Prefrontal Cortex
ECL: Evaluative Conditional Learning
EST: Emotional Stroop Task
FDA: Food and Drug Administration
MDI: Major Depression Inventory
MSQ: Mini Sleep Questionnaire
NfTM: Neurofunctional Tinnitus Model
PEI: positive emotion induction
STAI-S6: State Trait Anxiety Inventroy Small Questions
TBF-12: Tinnitus Impairment Questionnaire
tDCS: transcranial Direct Current Stimulation
THI: Tinnitus Handicap Inventory
TLQ: Tinnitus Loudness Questionnaire
TS: Tinnitus Severity
Vr: Valence-ratio.
