# Supplementary material for: Dose-Response Transcranial Electrical Stimulation Study Design: A Well-Controlled Adaptive Seamless Bayesian Method to Illuminate Negative Valence Role in Tinnitus Perception
Source: Front Hum Neurosci. 2022 May 12;16:811550. doi: 10.3389/fnhum.2022.811550 (PMC9169505; doi:10.3389/fnhum.2022.811550)
Supplement: Supplementary file 1 [file Data_Sheet_1.pdf]

Supplementary Material

1 Audiological and Tinnitus Psychoacoustic evaluation

Audiometry: Pure Tone Audiometry (125 - 16000 Hz) was applied for all participants to identify the level of their hearing loss.

Laterality: Tinnitus sound might be perceived unilaterally (right or left side) or bilaterally (equally both side or one side louder). This stage is crucial to determine which side is dominant in perceiving tinnitus (ipsilateral). Therefore, the non-dominant side (contralateral) was selected for psychoacoustic evaluations.

Similarity: The subjects were asked/examined to indicate the most similar tone to the perceiving tinnitus between the presented tones, including pure tone, narrow-band noise (NBN), Fresh noise (FN), warble tones, and white noise. The testing tones were centralized at 1 kHz frequency with 50 Decibel Hearing Level (HL) in loudness. The intensity got elevated when affected by hearing loss.

Pitch Matching test (PMT): The resembled tone was presented contra-laterally in ascending frequencies through a forced-choice method among two alternatives [3]. Based on the responses, the unselected alternative was replaced with a higher frequency step in range (125-16000 Hz) while the lower frequency was selected. Octave confusion (upper and lower) were also tested on the finally-selected frequency to confirm the matched pitch.

Testing Tone (TT): FN was regularly applied at the matched pitch to the dominant ear to measure the loudness-related parameters unless the tinnitus-resembling tone was recognized as FN. Thus, NBN was utilized alternately to prevent any confusion. TT was applied ascendingly for measuring

the following parameters:

Hearing Threshold Level (HTL): It was determined with the first perceived level of TT.

Loudness Match Test (LMT): Tinnitus loudness was identified by matching the TT with tinnitus level.

Minimal Masking Level (MML): It was marked as the lowest level of TT that masks tinnitus and makes it momentarily inaudible.

loudness discomfort level (LDL): It was defined as the most tolerable loudness level of TT.

2 Word lists in Portuguese and English translated

**Portuguese Version**	**English Version**
Garrafa	Bottle
Banheiro	WC
Luz	Light
Antigo	Old one
Milho	Corn
Patio	Courtyard

Table 2.1: Neutral World List

**Portuguese Version**	**English Version**
Cigarra	Cicada
Campainha	Bell
Tom	Tone
Apito	Whistle
Grilo	cricket
Radio	Radio

Table 2.2: Emotional World List

3 Dictionary and Acronyms

3.1 Dictionary

Placebo: a harmless pill, medicine, or procedure prescribed more for the psychological benefit to the patient than for any physiological effect.

- **Sham Stimulation (Placebo-like)**: "it consists in delivering an active stimulation for a few seconds to mimic the sensations observed with active tDCS and keep participants blind to the intervention"[2]. However, sham-controlled tDCS studies have reported inconsistent results from sham inconsistencies [8, 1, 9, 7, 6].

In this paper, placebo-like and sham trials were used interchangeably.

Seamless adaptive Design:Seamless designs incorporate both dose selection and confirmation of the efficacy of a selected dose, based on data from the entire trial[5].

Surrogate Endpoint: A surrogate endpoint is a clinical trial endpoint used as a substitute for a direct measure of how a patient feels, functions, or survives. A surrogate endpoint does not measure the clinical benefit of primary interest in and of itself, but rather is expected to predict that clinical benefit [4].

3.2 Acronyms

- Cognitive-Behavioral Model (CBM)
- Hearing Threshold Level (HTL)
- Loudness Match Test (LMT)
- Minimal Masking Level (MML)
- Pitch Matching test (PMT)
- Discomfort Level (DL)
- Tinnitus Handicap Inventory (THI)
- Fresh noise (FN)
- Hearing Level (HL)
- Testing Tone (TT)

- Tinnitus Impairment Questionnaire (TBF-12)
- Major Depression Inventory (MDI)
- State Trait Anxiety Inventroy Small Questions (STAI-S6)
- Clinical Global Impression (CGI)
- Mini Sleep Questionnaire (MSQ)
- Electroencephalogeraphy (EEG)
- Electronic Geodesic Incorporation (EGI)
- Region Of Interest (ROI)
- Dorsolateral Prefrontal Cortex (dlPFC)
- Emotional Stroop Task (EST)
- Neurofunctional Tinnitus Model (NfTM)
- Evaluative Conditional Learning (ECL)
- pre-frontal cortex (PFC)
- transcranial Electrical Stimulation (tES)
- Loudness Misperception Correction (LMC)
- transcranial Direct Current Stimulation (tDCS)
- High Definition (HD)
- Tinnitus Loudness Questionnaire (TLQ)
- Pure-tone audiometry (PTA)
- narrow-band noise (NBN)
- loudness discomfort level (LDL)
- Tinnitus Sample Case History Questionnaire (TSCHQ)
- Tinnitus Severity (TS)
- functional Magnetic Resonance Imaging (fMRI)
- Brodmann areas (BA)
- computer-aided design (CAD)
- Finite element (FE)

- electric field (EF)
- Conditional Stimulus (CS)
- Unconditional Stimulus (US)
- Nencki Affective Picture System (NAPS)
- arousal-ratio (Ar)
- Valence-ratio (Vr)
- resting-state (rs)
- event-related potential (ERP)
- Late Positive Potential (LPP)
- high-arousal-valence pictures (HAV)
- high-valence Pictures (HV)
- Food and Drug Administration (FDA)
- positive emotion induction (PEI)
- Minimum Clinical Efficacy (δ)
- "Sham concurrent with PEI" ("SP")
- "tDCS concurrent with PEI" ("tP") Coefficient of Variance (CV)
- Adaptive-Seamless Bayesian (ASB)
- Conscious Attended-Awareness Perception (CAAP)
- Non-invasive Brain Stimulation (NIBS)

REFERENCES

[1] Ammann, C., Lindquist, M.A., Celnik, P.A., 2017. Response variability of different anodal transcranial direct current stimulation intensities across multiple sessions. Brain stimulation 10, 757–763.

[2] Fonteneau, C., Mondino, M., Arns, M., Baeken, C., Bikson, M., Brunoni, A.R., Burke, M.J., Neuvonen, T., Padberg, F., Pascual-Leone, A., et al., 2019. Sham tdcs: A hidden source of variability? reflections for further blinded, controlled trials. Brain stimulation 12, 668–673.

[3] Goldstein, B., Shulman, A., 1991. Tinnitus evaluation. Tinnitus Diagnosis/Treatment. Philadelphia: Lea & Febiger , 293–318.

[4] Group, F.N.B.W., et al., 2016. Best (biomarkers, endpoints, and other tools) resource [internet] .

[5] GUIDANCE, D., 2018. Adaptive designs for clinical trials of drugs and biologics. Center for Biologics Evaluation and Research (CBER) .

[6] Lefaucheur, J.P., Antal, A., Ayache, S.S., Benninger, D.H., Brunelin, J., Cogiamanian, F., Cotelli, M., De Ridder, D., Ferrucci, R., Langguth, B., et al., 2017. Evidence-based guidelines on the therapeutic use of transcranial direct current stimulation (tdcs). Clinical Neurophysiology 128, 56–92.

[7] Martin, D.M., Moffa, A., Nikolin, S., Bennabi, D., Brunoni, A.R., Flannery, W., Haffen, E., McClintock, S.M., Moreno, M.L., Padberg, F., et al., 2018. Cognitive effects of transcranial direct current stimulation treatment in patients with major depressive disorder: an individual patient data meta-analysis of randomised, sham-controlled trials. Neuroscience & Biobehavioral Reviews 90, 137–145.

[8] Palm, U., Reisinger, E., Keeser, D., Kuo, M.F., Pogarell, O., Leicht, G., Mulert, C., Nitsche, M.A., Padberg, F., 2013. Evaluation of sham transcranial direct current stimulation for randomized, placebo-controlled clinical trials. Brain stimulation 6, 690–695.

[9] Silva, R.d.M.F.d., Brunoni, A.R., Miguel, E.C., Shavitt, R.G., 2016. Transcranial direct current stimulation for treatment-resistant obsessive-compulsive disorder: report on two cases and proposal for a randomized, sham-controlled trial. Sao Paulo medical journal 134, 446–450.
